# HLA-G1^+^ Expression in GGTA1KO Pigs Suppresses Human and Monkey Anti-Pig T, B and NK Cell Responses

**DOI:** 10.3389/fimmu.2021.730545

**Published:** 2021-09-09

**Authors:** Joseph Sushil Rao, Nora Hosny, Ramesh Kumbha, Raza Ali Naqvi, Amar Singh, Zachary Swanson, Heather Levy, Anders W. Matson, Magie Steinhoff, Nicole Forneris, Eric Walters, Bernhard J. Hering, Christopher Burlak

**Affiliations:** ^1^Department of Surgery, Schulze Diabetes Institute, University of Minnesota, Minneapolis, MN, United States; ^2^Division of Solid Organ Transplantation, Department of Surgery, University of Minnesota, Minneapolis, MN, United States; ^3^Medical Biochemistry and Molecular Biology Department, Suez Canal University, Faculty of Medicine, Ismailia, Egypt; ^4^Independent Consultant, Centralia, MO, United States

**Keywords:** HLAG-1, CRIPSR/Cas9, porcine, islet, xenotransplantation

## Abstract

The human leukocyte antigen G1 (HLA-G1), a non-classical class I major histocompatibility complex (MHC-I) protein, is a potent immunomodulatory molecule at the maternal/fetal interface and other environments to regulate the cellular immune response. We created GGTA1^-^/HLAG1^+^ pigs to explore their use as organ and cell donors that may extend xenograft survival and function in both preclinical nonhuman primate (NHP) models and future clinical trials. In the present study, HLA-G1 was expressed from the porcine ROSA26 locus by homology directed repair (HDR) mediated knock-in (KI) with simultaneous deletion of α-1-3-galactotransferase gene (GGTA1; GTKO) using the clustered regularly interspersed palindromic repeats (CRISPR)/CRISPR associated protein 9 (Cas9) (CRISPR/Cas9) gene-editing system. GTKO/HLAG1^+^ pigs showing immune inhibitory functions were generated through somatic cell nuclear transfer (SCNT). The presence of HLA-G1 at the ROSA26 locus and the deletion of GGTA1 were confirmed by next generation sequencing (NGS) and Sanger’s sequencing. Fibroblasts from piglets, biopsies from transplantable organs, and islets were positive for HLA-G1 expression by confocal microscopy, flow cytometry, or q-PCR. The expression of cell surface HLA-G1 molecule associated with endogenous β2-microglobulin (β2m) was confirmed by staining genetically engineered cells with fluorescently labeled recombinant ILT2 protein. Fibroblasts obtained from GTKO/HLAG1^+^ pigs were shown to modulate the immune response by lowering IFN-γ production by T cells and proliferation of CD4^+^ and CD8^+^ T cells, B cells and natural killer (NK) cells, as well as by augmenting phosphorylation of Src homology region 2 domain-containing phosphatase-2 (SHP-2), which plays a central role in immune suppression. Islets isolated from GTKO/HLA-G1^+^ genetically engineered pigs and transplanted into streptozotocin-diabetic nude mice restored normoglycemia, suggesting that the expression of HLA-G1 did not interfere with their ability to reverse diabetes. The findings presented here suggest that the HLA-G1^+^ transgene can be stably expressed from the ROSA26 locus of non-fetal maternal tissue at the cell surface. By providing an immunomodulatory signal, expression of HLA-G1^+^ may extend survival of porcine pancreatic islet and organ xenografts.

## Introduction

The prevention of antibody-mediated hyperacute rejection of organ xenografts in nonhuman primates (NHPs) through the disruption of the GGTA1 gene and/or the expression of human complement regulatory proteins in genetically engineered donor pigs has marked a major milestone in the development of xenotransplantation (1, 2). The cellular immune response to cell and organ xenografts is also vigorous (3–6) and genetic engineering of donor pigs to mitigate anti-pig cellular immunity has emerged as an important goal in the field (7–11).

In mice, NHPs and humans, cellular rejection of porcine xenografts is in part mediated by CD8^+^ T cells, with their direct priming by swine leukocyte antigen class I (SLA-I) playing an important role in the elicitation of cellular immunity (12, 13). Xenograft rejection also involves graft infiltration by NK cells (14). NHP NK cells become activated when in contact with porcine endothelial cells expressing SLA-I but suppressed when a human leukocyte antigen (HLA) is co-expressed (14, 15). Killer cell immunoglobulin-like receptors (KIR) on NK cells are specific for MHC-I molecules and have been demonstrated to downregulate cellular activation; however, the sequence and structural dissimilarities in MHC between pigs and humans are likely responsible for the lack of negative signaling to human NK cells (14–18). Development of porcine cells and organs expressing immunomodulatory molecules capable of modulating the cytotoxic actions of T and NK cells may make xenografts less susceptible to cellular rejection.

HLA-G is a nonclassical MHC molecule with immunomodulatory properties that protect the fetus from maternal immune responses, modulate both innate and adaptive immunity, maintain tolerance to self-antigens, enhancing transplant survival and promote immune escape in cancer and infectious diseases (19). Seven isoforms of HLA-G, including HLA-G1 (UNIPROT P17693), are expressed by villous cytotrophoblast and syncytiotrophoblast layers of the placenta, which lack expression of classical MHC class I or class II (20, 21). In spite of a short cytoplasmic tail, HLA-G1 is highly stable, accumulates on the cell surface, and has not been reported to undergo endocytosis (20). Considering that there are two free cysteine residues (Cys-42 and Cys-147) that render low allelic variation and restrict the peptide repertoire (22), the expression of HLA-G1^+^ on extra-villous trophoblasts (EVT) and placental cells provides a persistent and potent immunomodulatory signal (22, 23) that if expressed in other tissues may elicit a similar effect.

NK cells and macrophages constitute the majority (up to 70%) of immune cells in the uterine mucosa, luteal and secretory phase endometrium that come into contact with HLA-G1 (24). Most of the remaining leukocyte populations in the decidua are CD14^+^ macrophages (20–30%) and CD3^+^ T cells (5–15%) (25–27). These immune cells express high affinity cell surface receptors such as leukocyte Ig-like receptors (LILR), LILRB1 and LILRB2 [formerly known as Ig-like transcripts (ILT2 and ILT4)], which, when bound by HLA-G1, recruit intracellular phosphatases that de-phosphorylate the cytoplasmic domains of immune activation molecules such as the T cell or B cell receptors (28–31). The HLA-G1 molecules assemble on the cell surface to form a disulfide-linked dimer exhibiting an oblique configuration, exposing two upward facing binding sites on the α3 domain of the HLA-G1^+^ heavy chain with high affinity towards the aforementioned receptors (22). These LILRs are expressed on the cell surface of peripheral blood T and B lymphocytes, NK cells, and monocytes (32) and could potentially be suppressed in the xenograft environment if HLA-G1 were expressed on the graft. The ILT2 receptor expressed on B cells, NK cells, monocytes/dendritic cells, as well as subtypes of T cells, has a preference for HLA-G1 when associated with β2m (33).

Interaction with HLA-G1 at the cell surface inhibits the cytotoxic activity of B and T lymphocytes and NK cells (34–37), which are essential effector cells implicated in graft rejection (38). The presence of the smaller secreted form, HLA-G5^+^, expressed during heart, lung and kidney transplantation has been associated with improved allograft acceptance (39–41). In addition, CD4^+^HLA-G^+^IL-10^+^ cells have been described as a new population of HLA-G1^+^ expressing regulatory T cells that influence graft acceptance (42). Therefore, transgenic expression of HLA-G1^+^ on the cell or organ surface may protect xenografts from recipient B, T, NK cell and macrophage mediated rejection.

Miyagawa et al. described a HLA-G1 construct incorporating the cDNA of human β2-microglobulin (hβ2m) cloned from JEG-3 cell mRNA. The cDNAs were subcloned into the site of pCNX2 and the transcription of the inserted cDNA is driven by the β-actin promoter and the cytomegalovirus enhancer (11). Matsunami et al. used the cDNA of HLA-G1 and G3 with a FLAG epitope following the signal sequence of HLA-G1 and G3. HLA-G3 was thought to be easier to express on the cell surface than HLA-G1 due to its shorter length of its cDNA. However, the stable clones that expressed mRNA had a lower HLA-G3 expression than HLA-G1. Protein mis-folding of HLA-G3 in their study may have led to an unstable molecule and eventually translocated back into the cytosol to be degraded (43). HLA-E, while similar to HLA-G but not identical in function, was successfully expressed with the 15-kb genomic hβ2m fragment to create genetically engineered pigs (9).

In the present study, our goal was the transgenic expression of the HLA-G1 molecule in the porcine donor to form an HLA-G1-β2m complex capable of reducing activation and proliferation of human and monkey T, B, NK cells, and macrophages. Our strategy for the expression of HLA-G1 in pigs was predicated on the assumption that transgenic HLA-G1 associates with the endogenously expressed β2m protein to provide an immunomodulatory signal when interacting with the ILT2 receptor (33). We targeted the ROSA26 gene site as a safe harbor locus to constitutively express HLA-G1, which resulted in expression in all major transplantable organs and pancreatic islets. Genetically engineered islets isolated from these pigs restored normoglycemia in streptozotocin (STZ)-induced diabetic mice, suggesting that the insertion and expression of HLA-G1 or the deletion of the GGTA1 gene did not interfere with the reversal of diabetes. The demonstration of fluorescently-labeled ILT2 bound to the surface of porcine cells expressing HLA-G1 and the suppression of activation and proliferation of monkey and human B, T, and NK cells in coculture with HLA-G1-expressing cells suggested that the HLA-G1 expressed on cells of genetically engineered donors associated with endogenous β2m to create an immunomodulatory signal. It will be important to examine in future studies if the expression of HLA-G1 on xenografts can reduce the requirements for immunosuppression in xenotransplantation.

## Materials and Methods

### Ethical Statement

The handling and development of pigs were performed in accordance with the Federation of Animal Science Societies Guide for the Care and Use of Agricultural Animals in Research and Teaching, the United States Department of Agriculture Animal Welfare Act and Animal Welfare Regulations and were approved by the University of Missouri (NSRRC). Studies using mice for assessment of islet cell preparations and organ procurement in pigs were approved by the University of Minnesota Institutional Animal Care and Use Committee, conducted in compliance with the Animal Welfare Act (https://www.aphis.usda.gov/animalwelfare/downloads/AC_BlueBookAWA508_compversion.pdf) and adhered to principles stated in the Guide for Care and Use of Laboratory Animals (44).

### Cell Culture

Porcine fetal fibroblasts were grown in recovery media composed of Dulbecco’s Modified Eagle Medium (DMEM) supplemented with 20% fetal bovine serum (FBS), 1% Penicillin-Streptomycin Antibiotics and 100X 1% GlutaMAX™-I (Gibco) for 48 hours. After 48 hours, recovery media was exchanged for standard media composed of 10% FBS in DMEM media with 1% Penicillin-Streptomycin Antibiotics and 1% 100X GlutaMAX™-I. Both porcine and human cell lines (Jeg3, ATCC) were cultured in 10% FBS in DMEM media with 1% Penicillin-Streptomycin Antibiotics and 1% 100X GlutaMAX™-I. Deidentified blood samples were obtained through Memorial Blood Center (Saint Paul, MN) for peripheral blood mononuclear cells (PBMCs) isolation upon arrival.

### Optimal gRNA Design for Simultaneous GGTA1 Knock Out/ROSA26 Knock In and Evaluation of *In Vitro* Cleavage Potential

The guide RNA targeting ROSA26 was designed against the sequence GCCGGGGCCGCCTAGAGAAG in exon 1 to allow the intrinsic promoter to drive expression of HLA-G1^+^ at low to moderate levels to avoid cellular toxicity. Following confirmation of cleavage, gRNAs were evaluated for off target binding using the online ZiFiT Targeting software. Similarly, *GGTA1* specific gRNA, ATCTGCAAATACATACTTCA, were designed toward exon 9 containing the catalytic domain of the GGTA1 enzyme. Guide RNA sequences were ligated into px330 plasmids (Addgene) and propagated following manufacturer’s instructions.

Amplification of ~2000 kb sequence of the target genome, including sites for designed gRNAs, was performed using asymmetric primers, such that *in vitro* cleavage would result in two fragments of different sizes. High-yield PCR EcoDry premix was used to amplify the ROSA26 target amplicon. The following were added to the premix in a 0.2 ml PCR tube: 100 ng of porcine genomic DNA, 20 pm of forward and reverse primers (**Supplemental Table 1**), and 20 μl of nuclease free water. The following primer sequences were used (5’-3’): Left Forward: GCAGCCATCTGAGATAGGAACCCTGAAAACGAGAGG; Right Reverse: ATGCGCCTCCCACCCACAAGC.

Amplified product was loaded onto SYBR™ Green Nucleic Acid Gel Stain (Invitrogen) and desired bands were eluted by gel purification spin column. Briefly, 100 ng of experimental cleavage template was mixed with the components provided for *in vitro* CRISPR/Cas9 reaction, including 1 μl of Electroporation-Ready Guide-it Recombinant Cas9 endonuclease, 1 μl of 10X Cas9 Nuclease Reaction Buffer Buffer, and 1 µl of 10X bovine serum albumin (BSA) (Thermo-Fisher Scientific). The resulting mixture was diluted to a volume of 10 ul with nuclease free water and 20 nm ROSA26 sgRNA was added prior to incubation at 37°C for 1 hour. The reaction was halted by incubation at 70°C for 10 min. Reactions were analyzed on a 1% agarose gel alongside a negative control consisting of 100 ng of uncleaved ~2 kb control fragment.

### Overlapping PCR and HDR Assembly (HLA-G1 ROSA26 Template)

In order to achieve HLA-G1 insertion at exon 1 of the porcine ROSA26 locus, a HDR fragment was designed to include a 1000 bp sequence at the 5’ and 3’ ends of the target site. The HLA-G1^+^ sequence was codon optimized for *S. scrofa* gene expression. Based on alignment of sparsely available sequences for the promoter and exon 1, long-range PCR was performed according to the LongAmp^®^ *Taq* DNA Polymerase protocol and sequence was confirmed by next generation sequencing (NGS). The target sequence of the gRNA designed for ROSA26 was subsequently replaced by the *S. scrofa* codon optimized HLA-G1 sequence. The above components were assembled in a single HDR using overlapping PCR. Left and right fragments were amplified with an overlap of 50 bp using the synthesized HLA-G1 cDNA fragment. The overlapping region was allowed to anneal prior to extension by DNA polymerase, thereby creating a small amount of template, and PCR reaction was then performed by forward and reverse primer to amplify the left and right fragments of ROSA26, located 1000 bp left and right of the gRNA target site, respectively. Assembled product was gel purified using the gel purification spin column, according to manufacturer instructions.

### Cell Culture, Electroporation and Flow Sorting

Cryopreserved Mangalitsa pig fetal fibroblasts (PFF) were allowed to thaw at 37°C, washed twice with complete 10% Dulbecco’s Modified Eagle’s Medium (DMEM) (Life Technologies), and 2 x 10^6^ cells per petri dish were subsequently placed in 10% complete DMEM media. Media was changed every 48 hours to allow for at least 70% confluence. Cells were detached with Tryple Express (Life Technologies) 1X TrypLE™ Express Enzyme (Thermo-Fisher Scientific) and prepared for transfection, as per the Amaxa™ 4D-Nucleofector™ Protocol. In summary, 5 x 10^5^ cells were suspended in 75 μl transfection buffer prepared by mixing 82 μl Nucleofector™ Solution and 18 μl Nucleofector™ Supplement provided in the kit, as per manufacturer instructions. The remaining 25 μl of transfection buffer was used to mix gRNA plasmid and HDR template. Following incubation, the mixture was combined with PFF cells and transferred to Nucleocuvette cuvettes. Cells were subsequently transfected by electroporation using program CM-137, according to manufacturer instructions. Following transfection, cuvettes were kept at 37°C for 10 minutes to allow for cell recovery prior to being transferred to petri dishes. Media was changed 48 hours after transfection. At 70% confluence, cells were sorted by flow cytometric cell sorting. Briefly, cells were detached by TrypLE Express and labeled with isolectin B_4_ (IB_4_)-APC lectin (Invitrogen) and anti-human HLA-G1^+^-PE (clone 87G) in flow buffer composed of DMEM 1% BSA containing 1mM CaCl_2_, prior to incubation for 30 minutes at 4°C in the absence of light. Identical temperature incubation and centrifugation steps were performed with unstained cells. After washing twice with flow buffer in a 15 ml tube, cells were suspended in flow buffer and sorted under aseptic conditions using a 130 μm nozzle.

### DNA/RNA Isolation

DNA obtained from sections of transgenic pig tail or tissues were isolated using the QIAmp Fast DNA Tissue Kit (Qiagen). In addition, DNA was isolated from 1000 flow sorted cells using the QIAmp DNA Micro Kit (Qiagen). The RNEasy Minikit (Qiagen) was used to isolate RNA from selected tissue sections and 10^6^ cultured porcine fibroblast cells derived from sections of transgenic pig tail, according to manufacturer instructions.

### Gene Expression Analysis

The real-time PCR amplification reactions were carried out following the Minimum Information for Publication of Quantitative Real-Time PCR Experiments (MIQE) (45) in final volumes of 20 µl using SsoAdvanced™ Universal SYBR (1725270) according to the manufacturer’s instructions with CFX 96 real time PCR detection system (Bio-Rad). All samples were analyzed in triplicate and product identity was confirmed by melting curve analysis. Beta-actin was used as housekeeping gene for normalization. Primers and products sizes are shown in **Supplemental Table 1**. Fold changes of HLA-G1 expression in each tissue relative to human JEG3 cells were estimated *via* the Livak method based on the cycle threshold (Ct) value as follows: relative expression = 2−ΔΔCt, where ΔΔCt = (Ct HLA-G – Ct ACTB) porcine tissue − (Ct HLA-G – Ct ACTB) JEG3.

### Live/Dead analysis

Acridine Orange and Propidium Iodide (AOPI) are commonly used dyes to label live and dead cells. Acridine orange is a cell-permeable nucleic acid binding dye that emits green fluorescence with an excitation/emission of 500/526 when bound to dsDNA and red fluorescence when bound to ssDNA or RNA (460/650). Propidium Iodide is a red-fluorescent nuclear and chromosome counterstain with an excitation/emission of 533/617 permeable only to dead cells. PI binds to DNA by intercalating between bases with little or no sequence preference. When cells are labelled with both AO and PI, the PI quenches the AO in all cells without an intact nuclear membrane, making all nucleated cells fluoresce green and all dead cells fluoresce red. Labeled cells were analyzed by immunofluorescent microscopy.

### miR-375 Assay

TRIzol™ LS Reagent (Invitrogen) was used to isolate RNA from culture supernatants, as per manufacturer instructions to perform qRT-PCR using the TaqMan Gene Expression Assay (Applied Biosystems) specific for the miR-375 sequence.

### Confocal Microscopy

Confocal microscopy was performed on frozen/fixed sections from WT and GE adult pigs or fibroblast cells grown from tail sections of WT and GE piglets. Samples were fixed to slides or sub-cultured in tissue culture treated two-well chamber slides, with 10^5^ fibroblasts plated in each well containing complete DMEM media composed of 10% Fetal Bovine Serum (FBS), Penicillin Streptomycin Solution (Pen-Strep) and GlutaMAX™ Supplement (Gibco). Samples were fixed in 4% paraformaldehyde for one hour in room temperature and blocked with PBS-1% BSA for another hour at 4°C. Cells were labeled with (IB_4_) from *Griffonia simplificifolia*, Alexa Fluor™ 647 conjugate (Invitrogen) and anti-human HLA-G1^+^-PE antibody (clone 87G) and mounted using ProLong Gold antifade mount with 4′,6-diamidino-2-phenylindole (DAPI) nuclear stain (Invitrogen). Confocal microscopy was performed using the Olympus FLUOVIEW FV3000 confocal laser scanning upright biological microscope.

### Western Blotting Analysis

In brief, tail sections from transgenic pigs were lysed in RIPA Lysis and Extraction Buffer (Thermo-Fisher Scientific) with protease inhibitors at 4°C. Following lysis, supernatants were obtained by centrifugation at 13,000 ×g for 15 minutes at 4°C. Protein concentration was determined by BCA assay (Thermo-Fisher Scientific) and 10 μg of protein was separated on SDS-PAGE gel. Separated proteins were transferred to PVDF membrane and blocked with Odyssey Blocking Buffer (TBS) for one hour at room temperature. Anti-HLA-G1 (clone 87G) and anti-β-actin (SigmaAldrich) were used to probe membrane. Goat anti-Mouse I_g_G IRDye 800CW Secondary Antibody (Licor Biosciences) was used to detect primary antibodies on membranes using an Odyssey Fc Dual-Mode Imaging System (Li-Cor Biosciences).

### *In Vitro* Co-Culture Assay

Freshly isolated Human or NHP PBMCs were prepared using ACK lysis buffer and labeled with carboxyfluorescein succinimidyl ester (CFSE) (Invitrogen). Subsequently, wild-type (WT) or genetically engineered (GE) fibroblasts were irradiated (with 30 Gy) to stop proliferation. The target fibroblasts were mixed with PBMCs at a 1:1, 10:1, or 100:1 ratio in a 96-well flat-bottom plate for 6 hours to measure SHP-2 phosphorylation, 48 hours to measure intracellular IFN-γ production, or for 6 days to assess proliferation. For signaling experiments, hPBMCs were incubated with ImmunoCult human CD3/CD28 T Cell Activator (Stemcell) and Pansorbin (Millipore Sigma) for 48 hours prior to the addition of porcine fibroblast cells. Cells were labeled with anti-CD3 (Invitrogen), anti-CD4 (Invitrogen), anti-CD8 (Invitrogen), anti-CD16 (Invitrogen), anti-CD20 (Invitrogen), anti-CD28 (Invitrogen), and anti-CD56 (Invitrogen) as per manufacturer instructions. The percentage of positive cells for a given marker were measured by flow cytometry using a BD FACSCanto II cytometer. Data were analyzed using FlowJo software version 10.0 (TreeStar). Assays were performed in triplicate and repeated on three different human or NHP blood donors.

### Detection of Cell Surface HLA-G1/ILT2 Interaction

To determine if HLA-G1 expressed on the surface was associated with endogenous β2m and hence capable of binding with ILT2 on target cells (33, 34), we used a recombinant ILT2 labeled with streptavidin AF647 (R&D Systems/Invitrogen) and prepared following the manufacturer’s instructions. The percentage of rILT2 positive HLA-G1 expressing porcine fibroblast cells were measured by flow cytometry using a BD FACSCanto II cytometer and compared to JEG3 cells which naturally express HLA-G1. Data was analyzed using FlowJo software version 10.0 (TreeStar). Assays were performed in triplicate.

### Islet Isolation and Characterization

Porcine pancreases from five donors 22.4 ± 9 months of age and weighing 303.6 ± 126lbs were recovered and processed as described previously (46). Isolated islets were cultured at 37°C in complete medium E199 (Mediatech) with 10% heat inactivated porcine serum. Porcine islet preparations (n=28) were further characterized for islet cell composition (47, 48). Islet viability was measured after 24 and 48 hour culture by fluorescein AOPI staining (49). Endotoxin level of the final islet product was characterized by the Charles River Endosafe method (50).

### Transplantation of Islets Into Diabetic Mice

SPF male congenitally athymic nude mice Crl : NU-Foxn1nu obtained from Charles River Laboratories (Wilmington, MA) were used to bioassay for *in vivo* islet potency. The mice were kept under SPF conditions using cages equipped with filter tops and absorbent bedding (7092 Teklad Corn Cob Bedding, Harlan Laboratories, Madison, WI) in a temperature-controlled environment (22 to 25°C) on a 12:12-h light:dark photoperiod. Mice were housed in groups of 2 to 4 per cage. Water was provided as libitum, and animals were fed irradiated rodent diet (Diet 2919 Teklad Global 19% Protein Rodent Diet, Harlan Laboratories). Diabetes was induced by administration of clinical-grade STZ (Zanosar, Sicor Pharmaceuticals, Irvine, CA, USA) intraperitoneally at a dose of 240 mg/kg IP (51). Blood glucose (BG) was measured by tail stick and mice with BG levels exceeding 300 mg/dl were considered diabetic. Insulin therapy (glargine; Lantus, Sanofi-Aventis US, Bridgewater, NJ) was initiated at a dose of 0.5 U every other day after 3 consecutive glucose measurements exceeding 300 mg/dl. The dose and frequency of insulin administration was increased or decreased in response to a combination of measured glucose and body weight, until the islet transplantation. Eight diabetic mice were transplanted, at 17 ± 10wk age and weighing approximately 25 g, with a duration of diabetes of 23 ± 13d. Mice that were transplanted with islets were administered NSAIDs 10 minutes before incision and thereafter for three post-operative days. Mice were anesthetized with inhalation isoflurane, and artificial tear ointment was applied. The left kidney was located by palpation, and the surgical area was sterile prepped with Chloraprep. After incision, kidney was visualized and externalized, and the renal capsule was elevated to create a space for the cultured (day 8 ± 1) porcine islets (2,000 IEQ) to be transplanted using PE-50 tubing attached to a Hamilton syringe. The islet pellet size in the PE-50 tubing was 8 ± 3mm. The kidney was gently replaced into the retroperitoneum and the incision closed in a normal fashion with absorbable suture (18, 26). Blood glucose and body weight of the mice were measured up to 45 days. If the blood glucose level was persistently less than 200 mg/dl after transplantation, the mice were considered normoglycemic. After approximately 30 days of normoglycemia, mice underwent planned nephrectomy of the graft kidney. Mice were anesthetized with inhalation isoflurane, and artificial tear ointment was applied. The left kidney was located by palpation, and the surgical area was sterile prepped with Chloraprep. An incision was made overlying the kidney at the previous incision site, and gently exposed using palpation. The renal vessels were ligated, and the kidney removed. The incision was closed in a normal fashion with absorbable suture. Kidneys from both study groups were preserved in 10% formalin for confocal analysis.

### Statistical Analysis

Statistical analyses were performed using IBM SPSS software (IBM Software Group’s Business Analytics Portfolio) or GraphPad Prism version 8. Significance was determined with one-way ANOVA comparing 2 groups. When comparing mean values from different numbers of donors, or mean values from only one condition, untailed Students T-tests were performed. Results were considered significant when p < 0.05 and extremely significant when p < 0.01 and have been represented with (*) in the figures.

## Results

### Recombinant HLA-G1 Blocks *In Vitro* Porcine Cell and Islet Destruction by Human PBMCs

**Figure 1:**
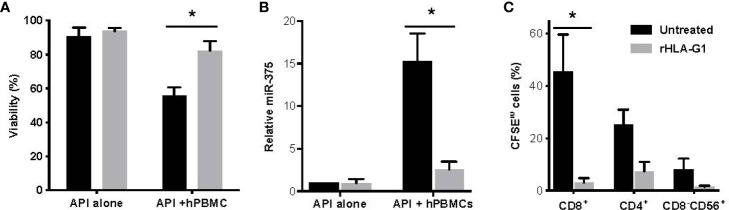
Adult porcine islets (API) protection from hPBMCs *in vitro* with anti-SLA or recombinant HLA-G treatment comparing untreated to rHLA-G1. **(A)** Comparative analysis of AO/PI viability staining of API treated with anti-SLA-I antibody (PT-85) with and without hPBMCs. **(B)** Relative release of miR-375 in culture supernatants of API treated with anti-SLA-I antibody. **(C)** Proliferation of CD4^+^ and CD8^+^ T cells and CD56^+^ NK cells with anti-SLA-I treatment of API. Proliferation was assessed by flow cytometry analysis of decreasing CFSE fluorescence. Co-cultures contained 100 IEQ/DNA of API (n = 2) and cultured with 10^5^ human PBMCs for five days. Data shown are presented as mean ± SD.

Recombinant HLA-G1 (rHLA-G1) inhibited the interaction of human B, T, and NK cells with adult pig islets (API) during co-culture with hPBMCs. The viability was measured by percent AO/PI staining. Viability of API was observed as 90.66% ± 3.51 for API alone, 52.66 ± 7.95 for API with hPBMCs, and 82.33 ± 9.29 for API with both hPBMCs and rHLA-G1^+^ treatment (p=0.0412) (**Figure 1A**). Following rHLA-G1^+^ treatment of API co-cultured with hPBMCs, miR-375 liberation fold values decreased from 15.01-fold ±1.44 to 2.52 fold ±0.26. rHLA-G1^+^ blocking resulted in a reduction of miR-375 in culture supernatant (p=0.0663) (**Figure 1B**). Treatment of mixed lymphocyte-islet co-culture with rHLA-G1^+^ significantly inhibited proliferation of CD4^+^ and CD8^+^ T cells (CD4, p=0.0653; CD8, p=0.0520; CD8-CD56+, p=0.164) (**Figure 1C**).

### Creation of HLA-G1 Template and gRNAs Specific to GGTA1 and ROSA26

The above findings suggested that generation of a transgenic pigs expressing the HLA-G1 gene from the porcine ROSA-26 locus using HDR-mediated KI by CRISPR/Cas9 gene editing would improve xenograft survival. We optimized the length of left and right homologous arms to improve KI efficiency at the porcine ROSA26 locus (52, 53). The sequence for synthesis of HDR and ROSA26 was first confirmed by Sanger sequencing. Previously published sequencing for the promoter region and exon 1 of the porcine ROSA26 locus was used to complete the gene template (54). The target sequence was interrogated against the porcine genome in the NCBI database using the Basic Local Alignment Search Tool (BLAST) (https://blast.ncbi.nlm.nih.gov/Blast.cgi) and a sequence similar to the NC_010455.4 (72802131 to 72802975) located at chromosome 13 in the *Sus scrofa* genome (*S. scrofa*). The left homologous arm of the HLA-G1 template was designed to include a 1000 base pair (bp) sequence spanning the promoter and exon 1 while a 1000 bp sequence located at the 3’ end was selected for design of the right homologous arm. Primers used to amplify the 1000 bp fragments by PCR are shown (**Supplemental Table 1**) and the resulting amplicon was sequenced by NGS. Interrogation of these sequences with the *S. scrofa* genome showed significant alignment.

The ZiFiT Targeter tool (http://zifit.partners.org/ZiFiT/) (55) was used to design the optimal guide RNA (gRNA) required to target exon 1 of the porcine ROSA26 locus. The gBlock of codon optimized HLA-G1 (produced by IDT) was added at the 3’ end in order to resolve sequence complexity for gBlock synthesis (**Supplemental Figure 1**). Left and right homologous fragments containing codon optimized and IDT modified HLA-G1^+^ were assembled with the HDR fragment using overlapping PCR. Primers for left and right homologous fragment HLA-G1 were designed such that reverse primer of left and forward primer of right fragment were overlapped by 50 bp with codon optimized HLA-G1 (**Supplemental Table 1**). Overlapping fragments were amplified individually by long-range PCR. Purified fragments were mixed for 30 cycles in PCR Master Mix (#K0171, Thermo-Fisher Scientific) without extension, prior to the addition of primer, Taq polymerase and deoxynucleotide (dNTP) for amplification of the assembled product (**Supplemental** **Figure 1**). The assembled product was subsequently eluted from gel and ready to be transfected in porcine fibroblasts. To delete the GGTA1 gene, gRNA was designed in the catalytic domain of exon 9 (**Supplemental Figure 1**).

### HLA-G1^+^ Porcine Fibroblasts Suppress Xenoreactive T and B Cell Proliferation

**Figure 2:**
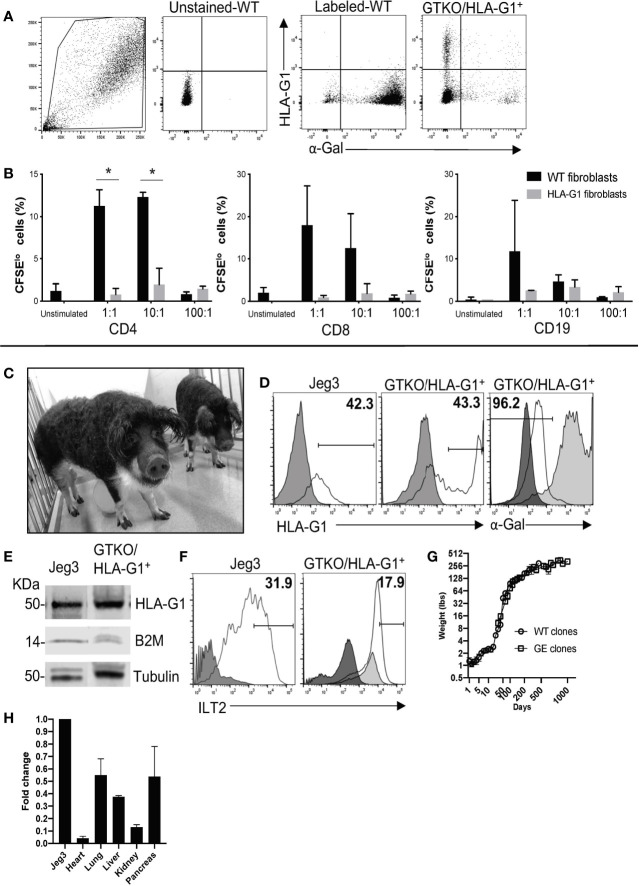
Creation and characterization of GTKO/HLA-G1^+^ pigs. **(A)** Flow sorting of GTKO/HLA-G^+^ cells beginning with forward and side scatter gate, unstained cell gating, untreated wild-type (WT) cell gating, and selected genetically engineered cells based on those gates. IB_4_ lectin and anti-HLA-G (clone 87G) monoclonal antibody were used to identify GGTA^-^ and HLA-G^+^ cells, respectively. **(B)** Sorted cells were examined for functional expression of HLA-G by co-culture with hPBMC (3 x 10^5^) at ratios of 1:1, 10:1, and 100:1 with sorted cells for five days. Data shown are presented as mean ± SD. Proliferation of CD4^+^ T cells, CD8^+^ T cells, and CD56^+^ NK cells by HLA-G^+^ GGTA1^-^ cells were measured by flow cytometry as a decrease in CFSE fluorescence. Prior to co-culture, hPBMCs were labeled with CFSE (2 µM), where dilution of CFSE was considered extent of proliferation. **(C)** Photograph of GTKO/HLA-G^+^ pigs generated by SCNT. **(D)** Expression of GGTA1 on skin fibroblasts derived from transgenic pigs (black line) compared to WT pig cells (medium grey) and unlabeled cells (dark grey). Human Jeg3 cells positive for HLA-G1 were used as a positive control. Unstained cells in dark grey were compared to anti-HLA-G labeled cells (clone 87G) shown as black line. Gates were based on the unstained cell fluorescence. **(E)** Western blot analysis of GTKO/HLA-G1^+^ transcript driven by the porcine ROSA26 promoter as detected with β2m and tubulin and compared to positive control Jeg3 cells. **(F)** Recombinant ILT2 bound to HLA-G1 positive porcine fibroblasts (black line) as compared to fluorescent streptavidin control (medium grey) and unlabeled cells (dark grey). JEG3 cells labeled with rILT2 were used as positive controls (black line) and compared to unlabeled cells (dark grey). **(G)** Comparison of weight gained by pigs, WT clones to GE clones, over a period of 1000 days of life. **(H)** Quantitative real time PCR of various transplantable organs from GTKO/HLA-G1^+^ to positive control JEG3 tissues demonstrating fold change corresponding to gene expression.

Porcine fibroblast cells were transfected with gRNAs specific for GGTA1 and ROSA26 and the linear assembled HDR template containing HLA-G1^+^ and sorted for HLA-G1^+^ and α-Gal^-^ cells using flow cytometry (FC) (**Figure 2A**). In order to evaluate the functions of HLA-G1^+^, HLA-G1^+^ porcine fibroblasts were co-cultured with hPBMCs at fibroblast:hPBMC ratios of 1:1, 10:1, and 100:1. A decrease in proliferating CD4^+^ and CD8^+^ T cells and CD19^+^ B cells was observed (**Figure 2B**). The proliferating fraction of CD4^+^ cells (i.e. of CD4^+^CFSE^lo^) for wild-type (WT) *vs.* HLA-G1^+^ fibroblast cells was found to be 11.24% ± 1.92 *vs.* 0.77% ± 0.72 (p=0.0188), 12.3% ± 0.56 *vs.* 1.96% ± 1.909188 (p=0.0180), and 0.82% ± 0.26 *vs.* 1.44% *vs.* 0.325 for 1:1, 10:1, and 100:1 ratios of fibroblast:PBMCs, respectively (**Figure 2B**). In addition, the CD19^+^CFSE^lo^ cell proportion was observed to be 11.75% ± 12.08 *vs.* 2.54% ± 0.056, 4.62% ± 1.59 *vs.* 3.37% ± 1.64, and 0.93% ± 0.155 *vs.* 2.125% ± 1.322 for fibroblast:hPBMC ratios of 1:1, 10:1, and 100:1, respectively (**Figure 2B**).

### Creation of GTKO and GTKO/HLAG^+^ Pigs

**Figure 3:**
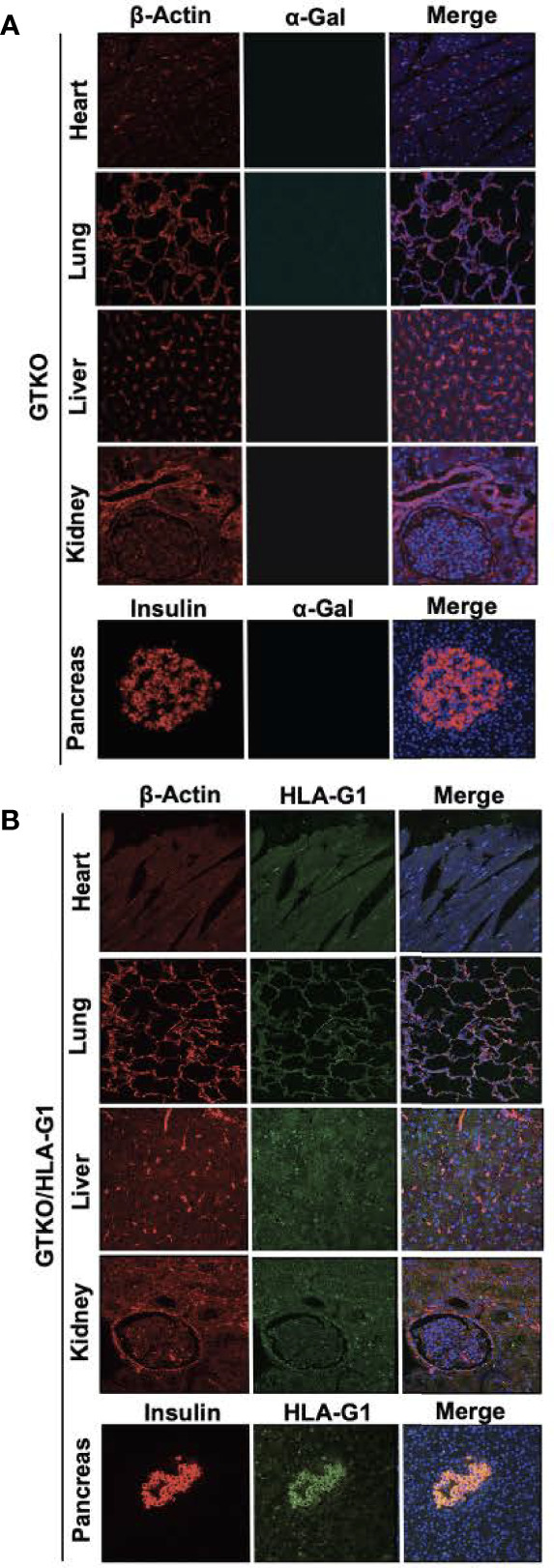
Confocal microscopy demonstrating physical characterization of genetically engineered tissues. **(A)** GTKO expression on heart, lung, liver, kidney and pancreas labelled with Rabbit mAb to b-actin [13E5] (Cell Signaling Technology; #4970S) and tagged to Goat anti-rabbit IgG AF 488 (Invitrogen; A11008), Isolectin GS-IB_4_ AF 647 Conjugate (Life Technologies; I32450) and DAPI. **(B)** GTKO/HLA-G1 on tissues was labelled using Mouse HLA-G monoclonal Antibody [87G] (Invitrogen; MA1-10356) and tagged to Goat anti-mouse IgG AF 555 (Invitrogen; A32727). Goat anti-mouse IgG1 Fc Secondary Antibody FITC (Invitrogen; 31547) was used as an isotype control for 87G. Pancreas section were labelled with Polyclonal Guinea Pig Anti-insulin (Dako; A0564) tagged to Donkey Anti-guinea pig IgG (H+L) AF 647 (Jackson Immuno Research; 706-606-148) instead of b-actin and DAPI. All sections were imaged using the 10X, 20X and 40X objective on Olympus Fluoview 3000 inverted confocal microscope.

GTKO/HLA-G1^+^ porcine fibroblasts were used for the generation of transgenic piglets by SCNT. A total of 211 cloned embryos were transferred to two surrogates, one of which exhibited full term-pregnancy and gave birth to five transgenic piglets (**Figure 2C** showing 2 of the 5 piglets). All five transgenic piglets were positive for the HDR fragment containing HLA-G1^+^ (**Figure 2D**). In addition to the specific ~3 kb band observed for HDR, a ~2 kb band was observed in both WT and GGTA1^-/-^/HLA-G1^+^ piglets, suggesting monoallelic integration of HLA-G1^+^. The correct integration of HLA-G1^+^ in the porcine ROSA26 locus was confirmed by NGS (**Supplemental Figure 1**). Sequencing for GGTA1 locus around the gRNA target site also showed the presence of mutations identical to those observed in gene edited cells prior to SCNT (**Supplemental Figure 1**). The HLA-G1^+^ transcript driven by the porcine ROSA26 promoter was confirmed by Western Blot analysis (**Figure 2E**). The demonstrated binding of rILT2 to 17.9% of cells prepared from GTKO/HLA-G^+^ pigs (**Figure 2F**), as compared to 31.9% of JEG3 cells, suggests that the expressed HLA-G1 dimerizes (or any other term that is better than associated with) with endogenous β2m. Engineered pigs followed a similar pattern of weight gain as that of cloned wild type pigs (**Figure 2G**). In addition, RNA was isolated from GGTA1^-/-^/HLA-G1^+^ piglets and HLA-G1^+^ specific TaqMan assay was performed to evaluate for the presence of HLA-G1 transcript driven by the porcine ROSA26 promoter with lung and pancreas having the highest fold change followed by liver, kidney and the heart. Real time PCR confirmed the presence of HLA-G1 and the absence of GGTA1 on the cell surface of fibroblasts obtained from GGTA1^-/-^/HLA-G1^+^ piglets (**Figure 2H**). Following procurement of organs from the pigs, paraffin embedded tissue sections were labelled with HLA-G1^+^ (Clone 87G), anti-beta-actin, isolectin (IB_4_) and DAPI. Immunofluorescence of the GTKO/HLA-G1^-^ sections confirm no expression of alpha-Gal on heart, lung, liver, kidney and pancreas (**Figure 3A**). GTKO/HLA-G1^+^ tissue sections show absence of alpha-Gal and expression of HLA-G1^+^ (**Figure 3B**). WT tissue sections used as controls however demonstrated no expression of HLA-G1^+^ and expressed alpha-Gal on their surface (**Supplemental Figure 2**).

### GTKO or GTKO/HLA-G1^+^ Islets Restore Normoglycemia

**Figure 4:**
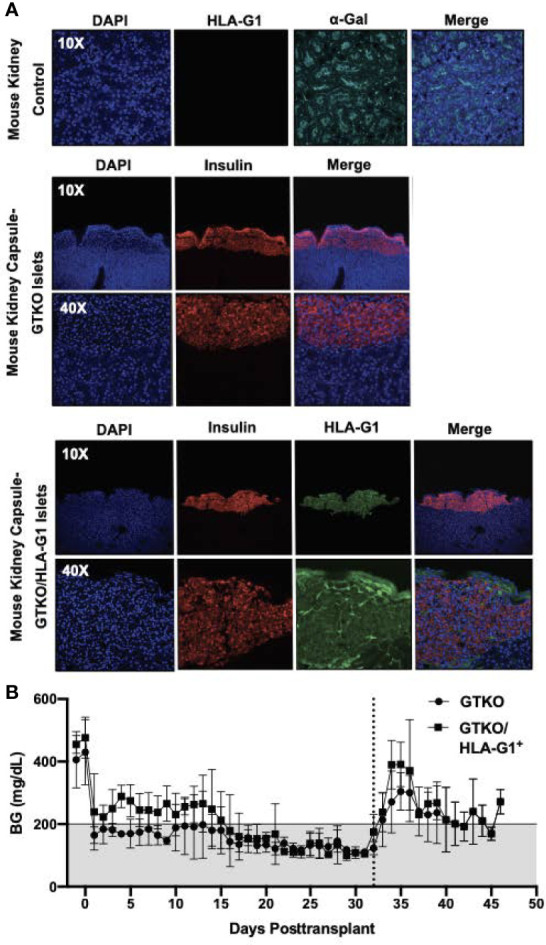
Kidney capsule islet transplantation characterization. **(A)** Mouse kidneys were paraffin embedded, sectioned and labelled with Isolectin GS-IB_4_ AF 647 Conjugate (Life Technologies; I32450), Mouse HLA-G monoclonal Antibody [87G] (Invitrogen; MA1-10356) tagged to Goat anti-mouse IgG AF 555 (Invitrogen; A32727) and DAPI as a control. The kidneys with the islet graft were labelled with Polyclonal Guinea Pig Anti-insulin (Dako; A0564) tagged to Donkey Anti-guinea pig IgG (H+L) AF 647 (Jackson Immuno Research; 706-606-148), Mouse HLA-G monoclonal Antibody [87G] (Invitrogen; MA1-10356) and tagged to Goat anti-mouse IgG AF 555 (Invitrogen; A32727). DAPI was used to label nuclei of all cells. Isolectin GS-IB_4_ was not used to avoid non-specific excitation. All sections were imaged using the 10X, 20X and 40X objective on Olympus Fluoview 3000 inverted confocal microscope. **(B)** GTKO or GTKO/HLA-G1 islets were transplanted under the kidney capsule of STZ-diabetic nude mice. Blood glucose measurements through Day 32 were used to determine normoglycemia.

A total of five genetically engineered donor pigs were used for islet isolation, including three GTKO and two GTKO/HLA-G1^+^. Seven day-cultured islets were transplanted under the kidney capsule of STZ-diabetic nude mice. Islet transplantation from the two GTKO/HLA-G1^+^ and three GTKO reversed diabetes in 3/3 and 5/5 mice, respectively. The proportions of beta cells in the transplanted islet preparations from the GTKO/HLA-G1^+^ and GTKO donor pigs were 61% and 74.5%, respectively. The kidney capsule demonstrated insulin secretion in GTKO/HLA-G1^-^ islets and colocalized HLA-G1^+^ on GTKO/HLA-G1^+^ islets (**Figure 4A**). Following transplantation, the shortest duration to restore normoglycemia posttransplant was one day. In the remaining recipients, normalization of blood glucose (BG) levels was demonstrated by day 16 posttransplant with minimal fluctuations, until a unilateral nephrectomy with the islet graft was performed on posttransplant day 32 to demonstrate reversal of diabetes. The rise in BG >200 mg/dl confirmed diabetes and the mice were euthanized thereafter (**Figure 4B**). The kidneys with the graft were paraffin embedded, sectioned and labelled with DAPI, anti-insulin antibody, anti-HLA-G1 antibody, and imaged using confocal microscopy. Sections of kidney without the islet graft were used to demonstrate alpha-Gal positivity and absence of HLA-G1 expression (**Figure 4A**).

### Porcine GTKO/HLA-G1^+^ Cells Suppress Cytokine, Proliferative and Signaling Responses in Xenogeneic PBMCs

**Figure 5:**
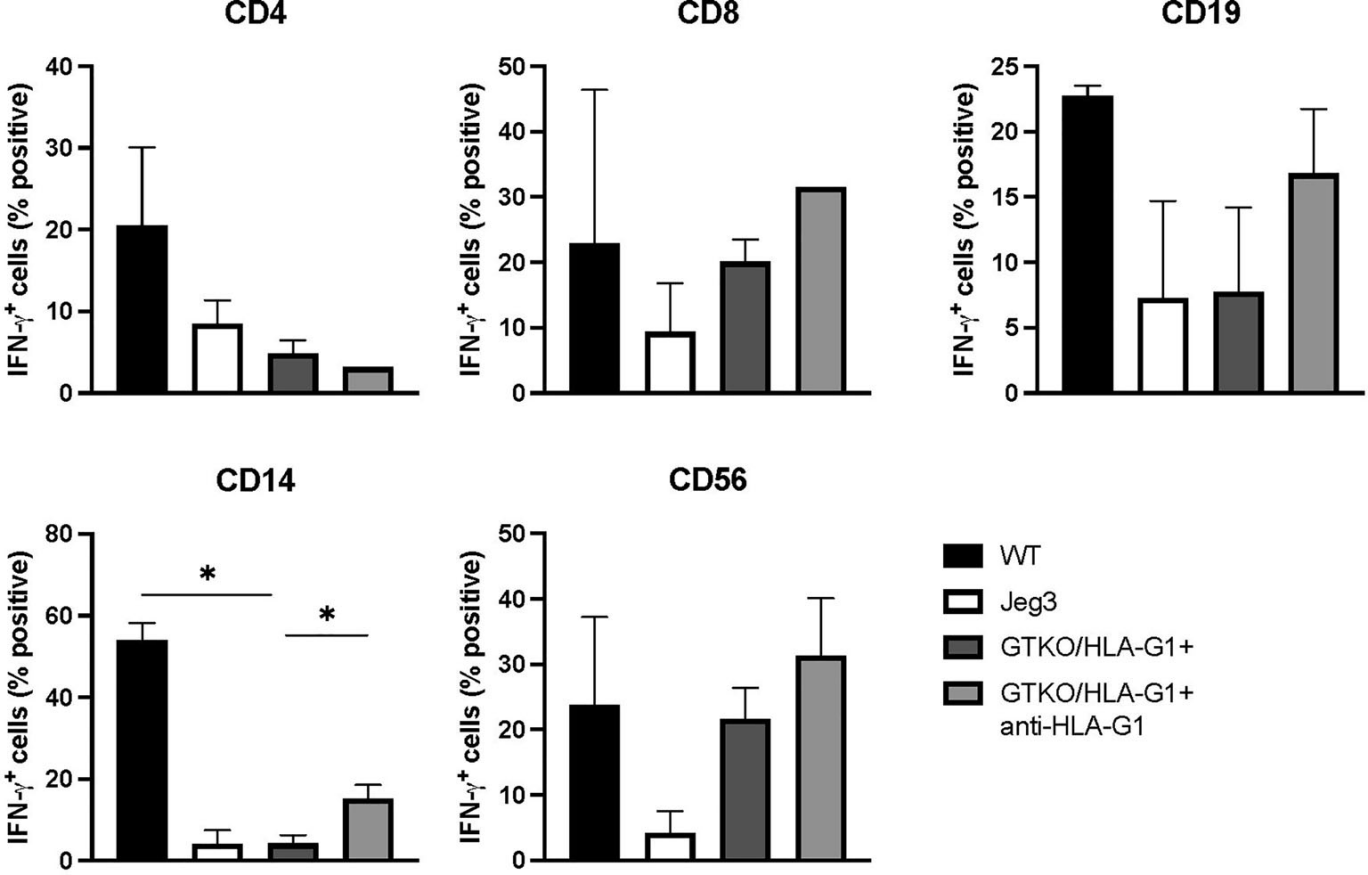
Transgenic porcine HLA-G^+^ cells dampen IFN-γ secretion from various subsets of human PBMCs in co-culture. Proportion of CD4^+^, CD8^+^ IFN-γ^+^ T cells; CD19^+^ IFN-γ^+^ subset; CD14^+^ IFN-γ^+^ macrophage subset; CD56^+^IFN-γ^+^ NK cell subset. All cultured cells were harvested after 48h. JEG-3 cells were used as positive controls for HLA-G expression. Data shown are presented as mean ± SD.

Fibroblasts isolated from GTKO/HLA-G1^+^ piglets were co-cultured with hPBMCs and responder cells were analyzed for intracellular IFN-γ staining. Controls included the use of fibroblasts from WT pigs and HLA-G1-expressing JEG-3 as well as the addition of anti-human HLA-G1^+^ blocking antibody to co-cultures with GTKO/HLA-G1^+^ fibroblasts and hPBMCs. Expression of HLA-G1^+^ reduced IFN-γ secretion by CD4^+^ and CD8^+^ T cells, CD14^+^ macrophages (WT: GTKO/HLA-G1^+^, p=0.0045), NK cells, and JEG-3 cells compared to WT fibroblasts (**Figures 5A–E**). Inversely, IFN-γ secretion increased upon anti-HLA-G1^+^ treatment (CD14^+^ macrophages, GTKO/HLA-G1^+^:GTKO/HLA-G1^+^ and anti-HLA-G1^+^ antibody, p=0.0574) (**Figure 5**).

**Figure 6:**
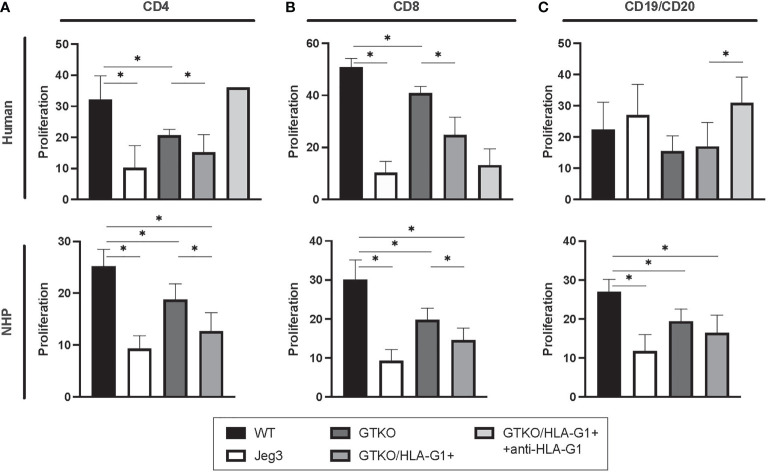
Amelioration of hPBMC proliferation *in vitro* by skin fibroblasts obtained from HLA-G^+^ transgenic pigs. **(A)** Proportion of CD4^+^CFSE^-^ T cells subset **(B)** Proportion of CD8^+^CFSE^-^ T cells subset **(C)** Proportion of CD19^+^CFSE^-^ subset. JEG3 cells were used as positive controls for HLA-G expression. Transgenic cells were treated with anti-HLA-G (87G) (5 µg/mL) for HLA-G blockade. Data shown are presented as mean ± SD.

Cells isolated from GTKO/HLA-G1^+^ pigs were capable of dampening the proliferation of human CD4^+^ (WT: GTKO, p=0.0004; WT : JEG3, p=0.0034; GTKO: GTKO/HLA-G1^+^, p=0.0136) and CD8^+^ T cells (WT: GTKO, p=<0.0001; WT : JEG3, p=<0.0001; GTKO: GTKO/HLA-G1^+^, p=<0.0001) as well as CD19^+^ or CD20^+^ B cells (WT: GTKO/HLA-G1^+^, p=0.1420; GTKO/HLA-G1^+^: GTKO/HLA-G1^+^+anti-HLA-G1^+^ antibody, p=0.0382) as measured by FC of reduced CFSE in cultured PBMCs (**Figure 6**). Cells isolated from GTKO/HLA-G1^+^ pigs were also capable of reducing the proliferation of monkey CD4^+^ (WT: GTKO, p=0.0006; WT : JEG3, p=<0.0001; GTKO: GTKO/HLA-G1^+^, p=0.0018; WT: GTKO/HLA-G1^+^, p=,0.0001) and CD8^+^ T cells (WT: GTKO, p=<0.0001; WT : JEG3, p=<0.0001; GTKO: GTKO/HLA-G1^+^, p=0.0018; WT: GTKO/HLA-G1^+^, p=<0.0001) as well as CD19^+^ or CD20^+^ B cells (WT : GTKO, p=0.0001; WT: GTKO/HLA-G1^+^, p=<0.0001; WT : JEG3, p=<0.0001; GTKO: GTKO/HLA-G1^+^, p=0.1267) as measured by FC of reduced CFSE in cultured PBMC (**Figure 6**). Anti-HLA-G1 antibody treatment in cocultures with human PBMC confirmed the impact of HLA-G1 expression by blocking the immune inhibitory effect.

**Figure 7:**
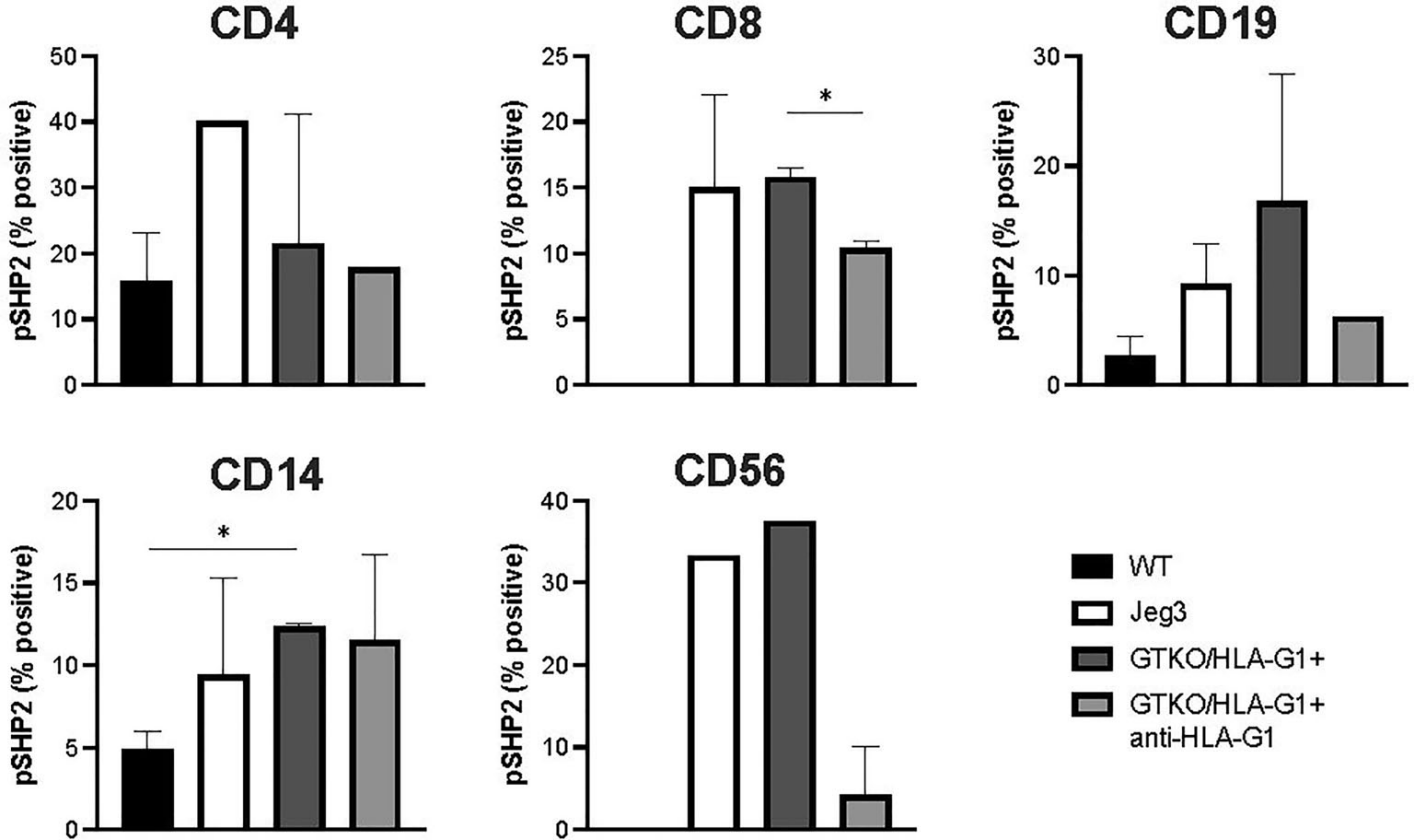
Skin fibroblasts derived from HLA-G^+^ porcine cells increase phosphorylation of SHP-2 in various subsets of human PBMCs. Proportion of CD4^+^, CD8^+^ SHP-2^+^ T cells subset; CD19^+^SHP-2^+^ subset; CD14^+^SHP-2^+^ B cells subset; CD56^+^ SHP-2^+^ NK cells subset. Prior to addition of HLA-G^+^ skin fibroblasts, hPBMCs were stimulated with anti-CD3/anti-CD28 cocktail for 72h, as per manufacturer instructions. In order to measure SHP-2 phosphorylation in B cells, hPBMCs were stimulated with pansorbin.

HLA-G1^+^ mediated phosphorylation of SHP-2 is one the key down-stream events leading to dephosphorylation of mammalian target of rapamycin (mTOR) in T cells and Akt in B cells (56) and in a dose dependent manner (57). In the present study, HLA-G1^+^ expressing porcine fibroblast cells collected from engineered piglets stimulated increased phosphorylation of SHP-2 in activated hPBMCs during coculture (**Figure 7** **)**. While there were observable differences in the measurement of pSHP2, only the signaling in CD8^+^ cells (GTKO/HLA-G1^+^: GTKO/HLA-G1^+^ + anti-HLA-G1 antibody, p=0.0138) and CD14^+^ macrophages (WT: GTKO/HLA-G1^+^, p=0.0101) were found to be significantly reduced by coculture with HLA-G1^+^ porcine fibroblast.

## Discussion

This study showed that expression of HLA-G1 in porcine cells suppressed the human and monkey T, B, and NK cell response *in vitro*. Using CRIPSR/Cas9, we deleted the GGTA1 gene to reduce anti-Gal antibody binding (58) and inserted the HLA-G1 gene in the ROSA26 site. We created and characterized GTKO/HLA-G1^+^ pigs, demonstrated ILT2 binding to HLA-G1^+^ cells, and suppression of activation and proliferation of human immune cells when co-cultured with HLA-G1-expressing cells, suggesting expression of an immunomodulatory HLA-G1 molecule. Since demonstrating efficacy in a preclinical NHP model will be a requirement for utilizing these engineered pigs as organ donors, we tested cynomolgus macaque immune cell responses to HLA-G1 expressing cells *in vitro*. Cells from GTKO/HLAG1^+^ pigs suppressed the activation and proliferation of monkey CD4^+^ and CD8^+^ T cells, B cells, macrophages and NK cells. Islets isolated from those pigs expressed HLA-G1^+^ and, when transplanted under the kidney capsule in diabetic mice, restored normoglycemia. Our results suggest that the insertion of the HLA-G1 gene in porcine donors could mitigate the cellular immune response to the xenograft.

Perhaps the best characterized effect of native HLA-G1 is in the cytotrophoblast layer of the placenta where there are approximately 70% NK cells (CD56^bright^), 10 to 20% antigen presenting cells (APCs) and T cells (59). The effect of HLA-G1 has been demonstrated to protect fetal cytotrophoblast cells from NK cell-mediated lysis (35). To better understand the role of HLA-G1 during xenotransplantation we added rHLA-G1 to co-cultures of adult procine islets and human PBMCs which resulted in significantly less release of miR-375 in culture supernatants and almost negligible PI-stained API regions. Release of miR-375 microRNA has been shown to be a useful biomarker for early graft rejection (55, 60). This suggested that HLA-G1 could protect API from hPBMC-mediated anti-porcine responses *in vitro*. The action of HLA-G1 is regulated by inhibitory receptor crosslinking (61) and therefore it was an important observation that supplemental rHLA-G1 could overcome the cellular activation imposed by the imperfect binding interaction of SLA class I and human NK inhibitory receptors (56, 57). Our initial observations of the role of rHLA-G1 to reduce human B, T, and NK cell proliferation suggested that insertion of the HLA-G1 gene if expressed on the cell surface and associated with the endogenous β2m may reduce the cellular attack directed towards xenografts.

Human HLA-G1 was successfully expressed from the porcine ROSA26 locus using CRISPR/Cas9-mediated KI gene editing. The sparsely available sequence of pig ROSA26 gene in NCBI database was confirmed by NGS prior to designing gRNA to target exon 1 in order to achieve successful HLA-G1 gene insertion. Maintaining the splicing sequence ruled out the possibility of HLA-G1 loss during ROSA26 mRNA splicing. In order to design the HDR template, several bases were removed from exon 1 of the porcine ROSA26 gene locus prior to insertion of *S. scrofa* codon optimized HLA-G1, including the gRNA target site. HLA-G1 expression could be further enhanced by optimizing the distance of insertion from the ROSA26 promoter region. However, natural promoter methylation regions may interfere with homologous arm association and gene insertion.

Using the GTKO/HLA-G1^+^ fibroblast cells, transgenic piglets were successfully generated and confirmed for the GTKO/HLA-G1^+^ genotype. Data obtained by NGS of DNA isolated from the tails of the GTKO/HLA-G1^+^ piglets confirmed the presence of HLA-G1^+^ in the porcine ROSA26 locus. Successful transcription of the inserted HLA-G1 gene by CRISPR/Cas9 gene editing was verified by either qRT-PCR or NGS. Furthermore, confocal microscopy of fibroblasts grown from the tails of transgenic piglets further confirmed the presence of HLA-G1 and absence of the GGTA1 gene. As expected, mature pigs showed HLA-G1 expression in all tissues evaluated.

The natural expression of HLA-G1^+^ has been measured in cardiac allograft recipient biopsies revealing 86% of HLA-G1^+^ grafts had no cellular rejection and correlated to a 0% risk of vasculopathy (39, 62). HLA-G1^+^ expression in kidney, liver and kidney/liver composite grafts was associated with the presence of immunosuppressive IL-10 expression, suggesting that transgenic expression of HLA-G1^+^ would directly regulate T, antigen-specific CTL, B, and NK cells and promote an environment of IL-10 driving maintenance of immune suppression (63–65). In our experiments, HLA-G1 mediated immune modulation of CD8^+^, CD4^+^, NK cells, macrophages, B cells occurred within the first 48h of culture. Our results are in agreement with previous research measuring the production of IFN-γ, indicating the proliferative response was inhibited according to the ratio of the HLA-G1^+^-expressing target cell population (61). Thus, HLA-G1 expression on xenografts may provide immediate immune protection and may contribute to long-term graft survival through immunosuppression of adaptive immune cell activation.

Transgenic HLA-G1 expression may provide a necessary regulatory signal for porcine xenografts because the potential porcine orthologues, non-classical SLA class Ib, 6,7, and 8, while functional, lack conserved sequences of HLA-G1^+^ required for inhibitory signaling through human ILT2 (T, B, NK, and DCs and monocytes), ILT4 (DCs and monocytes), and KIR2DL4 (NK cells) inhibitory receptors (28, 66, 67). Therefore, porcine expression of HLA-G1^+^ for xenotransplantation to monkeys for preclinical studies or to humans would be necessary to exert these immunosuppressive effects. The cognate receptors of HLA-G1, ILT2 and ILT4, are present in monkeys (NM_001040672.2 and XM_015441717.1, respectively) along with the HLA-G1 equivalent, *mafa-AG*, and are part of a functionally orthologous system of immune regulation (68–70). Among macaques and humans, ILT2 and ILT4 share ITIM domains and equivalent binding sites with high protein sequence homology, although minor glycosylation and SNPs have been identified (71). The suppression of monkey PBMCs in parallel experiments to human PBMCs in this study support the utility of this genetically engineered porcine donor in both preclinical studies and potential clinical trials. This was further supported in our study when recombinant ILT2 receptor (rILT2) was capable of binding a conformationally dependent cell surface HLA-G1, which suggested binding to endogenous porcine β2m (33, 34, 43).

Inhibition of IFN-γ production in macrophages and NK cells by membrane bound HLA-G1 are novel observations of this study. The HLA-G1 gene mediates negative regulatory signals through the immunoreceptor tyrosine-based inhibitory motif (ITIM) located in ILT2 and ILT4 on lymphocytes. In this study, lymphocytes exhibiting higher expression of ILT4, including CD4^+^ and CD8^+^ T cells, macrophages and NK cells, compared to those with higher expression of ILT2, i.e. B cells, were found to be the most affected. It is possible that the extent of ILT4 expression on these cells could explain the varying levels of HLA-G1 mediated suppression observed; however, more investigation into this area is needed. Ristich et al. demonstrated that human ILT4 receptors on dendritic cells stimulated by HLA-G1 contributed to the long term survival of allogeneic grafts (72). Crosslinking ILT4 downregulated MHC-Class II cell surface expression, costimulatory molecules and modulated cytokine production improving long term graft survival possibly through the induction of regulatory T cells and T-cell anergy. While we did not measure MHC class II cell surface expression in our study it is plausible that decreased expression of MHC class II and costimulatory molecules as observed by Ristich et al. may be the downstream mechanism by which HLA-G1 expression in our model mediated the immunosuppressive effects.

Multiple down-stream signaling molecules are responsible for orchestrating the suppressive effect of HLA-G1 interaction with activated human immune cells *in vitro* such as phosphorylation of SHP-2 (73, 74). Both SHP-1 and SHP-2 have central catalytic domains containing the protein-tyrosine phosphatase (PTP) signature motif, VHCSAGIGRTG, two Src-homology 2 (SH2) domains at their N-termini, and a C-terminus with potential tyrosine phosphorylation sites (75). To avoid autophosphorylation, both SH2 domains are intramolecularly associated with the single PTP domain. Tyrosine phosphorylation induces the active configuration of SHP-2, which, in turn, interacts with ILT2 and ILT4 receptors and further modulates the functioning of immune cells (28). We demonstrated that HLA-G1 expressed in pigs could signal to human T cells, B cells, macrophages, and NK cells phosphorylating SHP-2, but additional studies are necessary to shed light on downstream effects of HLA-G1 signaling in the context of xenotransplantation. Phosphorylation of SHP-2 was reduced upon anti-HLA-G1 treatment and the extent of HLA-G1^+^ induced SHP-2 phosphorylation in human immune cells was found to be similar to previously published data (56). Ketroussi et al. demonstrated that the chemical inhibitor of SHP-2, NSC-8787, precludes the downstream dephosphorylation of mTOR and arrest of the T cell cycle (73). In contrast to the soluble HLA-G1 used by Ketroussi et al. (73), the data presented in this study is based on membrane bound HLA-G1 and blocking with an anti-HLA-G1 antibody. Our observation that HLA-G1 induced SHP-2 phosphorylation in macrophages was not entirely unexpected (76). Earlier reports have demonstrated SHP-2 phosphorylation upon HLA-G1-ILT4 interaction and modulation of the nuclear factor kappa-light-chain enhancer of activated B cells (NF-κB) pathway (77). Whether the same mechanisms dominate during HLA-G1 mediated attenuation of macrophages remains to be studied.

It was not known if the ROSA26 gene site would drive expression of the HLA-G1 protein in islets or other organs relevant to transplantation. We confirmed the expression of HLA-G1 on the cell surface and observed protein in the cell cytoplasm as well. A limitation of this construct may be the finding that rILT2 bound to only 17.9% of HLA-G1^+^ cells versus 31.9% of JEG3 control cells. This difference may be due to the proximity of the HLA-G1 gene to the ROSA26 promoter or the affinity of the expressed HLA-G1 protein to the endogenous porcine β2m protein as compared to the human β2m expressed on JEG3 cells. The demonstrated restoration of normoglycemia in STZ-mice after transplantation of islets isolated from GTKO/HLA-G1 pigs suggests that the insertion of the HLA-G1 gene does not interfere with critical transplant function. Whether these findings extend to solid organ xenografts remains to be investigated. Future studies should consider optimization of the HLA-G1 construct to reduce intracellular protein that could be misfolded or incompletely synthesized.

Based on the above findings, we conclude that pigs cloned with the GTKO/HLA-G1^+^ phenotype had long-term viability and express a functionally active HLA-G1 capable of reducing anti-porcine immune responses from humans and NHPs. Fine tuning HLA-G1 expression to a threshold that will maximize the immunomodulatory effect may reduce the need for maintenance immune suppression in future xenotransplant recipients.

## Data Availability Statement

The raw data supporting the conclusions of this article will be made available by the authors, without undue reservation.

## Ethics Statement

The animal study was reviewed and approved by University of Missouri (NSRRC) and University of Minnesota Institutional Animal Care and Use Committees (IACUC) and Institutional Biosafety Committees. Written informed consent was obtained from the owners for the participation of their animals in this study.

## Author Contributions

JSR, NH, RK, and RN designed and executed experiments. AS, ZS, HL, AM, MS, and NF executed experiments. EW executed the creation and maintenance of genetically engineered pigs. BH provided creative guidance and critical review of the manuscript. CB conceived of the study, provided funding, designed experiments and was in charge of the overall direction and planning. JSR and CB wrote the manuscript. All authors contributed to the article and approved the submitted version.

## Funding

This study was funded by sponsored research agreements governed by the University of Minnesota with Diabetes Free, Inc. The funder was not involved in the study design, collection, analysis, interpretation of data, the writing of this article or the decision to submit it for publication.

## Conflict of Interest

BH has an equity interest in and serves as paid executive officer and director of Diabetes Free, Inc., an organization that may commercially benefit from the results of this research. This interest has been reviewed and managed by the University of Minnesota in accordance with its conflict of interest policies.

The remaining authors declare that the research was conducted in the absence of any commercial or financial relationships that could be construed as a potential conflict of interest.

## Publisher’s Note

All claims expressed in this article are solely those of the authors and do not necessarily represent those of their affiliated organizations, or those of the publisher, the editors and the reviewers. Any product that may be evaluated in this article, or claim that may be made by its manufacturer, is not guaranteed or endorsed by the publisher.

## Supplementary Material

Supplementary Figure 1 HLA-G1 integration in the porcine ROSA26 locus. **(A)** Sanger sequence product of GTKO/HLA-G1 piglet **(B)** Next generation sequence of HLA-G1^+^.

Supplementary Figure 2 Confocal analysis of WT porcine tissue samples. WT heart, lung, liver, kidney and pancreas were paraffin embedded, sectioned and labelled with Rabbit mAb to b-actin [13E5] (Cell Signaling Technology; #4970S) and tagged to Goat anti-rabbit IgG AF 488 (Invitrogen; A11008), Isolectin GS-IB_4_ AF 647 Conjugate (Life Technologies; I32450), Mouse HLA-G monoclonal Antibody [87G] (Invitrogen; MA1-10356) and tagged to Goat anti-mouse IgG AF 555 (Invitrogen; A32727). Goat anti-mouse IgG1 Fc Secondary Antibody FITC (Invitrogen; 31547) was used as an isotype control for 87G. Pancreas section were labelled with Polyclonal Guinea Pig Anti-insulin (Dako; A0564) tagged to Donkey Anti-guinea pig IgG (H+L) AF 647 (Jackson Immuno Research; 706-606-148) instead of b-actin. DAPI was used to label nuclei of all cells. All sections were imaged using the 10X, 20X and 40X objective on Olympus Fluoview 3000 inverted confocal microscope.

Supplementary Table 1 Quantitative PCR probe sequences and product size.

## References

[1] Lutz AJ Li P Estrada JL Sidner RA Chihara RK Downey SM. Double Knockout Pigs Deficient in N-Glycolylneuraminic Acid and Galactose Alpha-1,3-Galactose Reduce the Humoral Barrier to Xenotransplantation. Xenotransplantation (2013) 20(1):27–35. 10.1111/xen.12019 23384142

[2] Cowan PJ Chen CG Shinkel TA Fisicaro N Salvaris E Aminian A. Knock Out of Alpha1,3-Galactosyltransferase or Expression of Alpha1,2-Fucosyltransferase Further Protects CD55- and CD59-Expressing Mouse Hearts in an Ex Vivo Model of Xenograft Rejection. Transplantation (1998) 65(12):1599–604. 10.1097/00007890-199806270-00010 9665076

[3] Butler JR Wang ZY Martens GR Ladowski JM Li P Tector M. Modified Glycan Models of Pig-to-Human Xenotransplantation Do Not Enhance the Human-Anti-Pig T Cell Response. Transpl Immunol (2016) 35:47–51. 10.1016/j.trim.2016.02.001 26873419

[4] Wang ZY Morsi M Nguyen HQ Bikhet M Burnette K Ayares D. The Human T-Cell Proliferative Response to Triple-Knockout Pig Cells in Mixed Lymphocyte Reaction. Xenotransplantation (2020) 27(5):e12619. 10.1111/xen.12619 32529670 PMC7541622

[5] Ezzelarab M Ezzelarab C Wilhite T Kumar G Hara H Ayares D. Genetically-Modified Pig Mesenchymal Stromal Cells: Xenoantigenicity and Effect on Human T-Cell Xenoresponses. Xenotransplantation (2011) 18(3):183–95. 10.1111/j.1399-3089.2011.00635.x 21696448

[6] Wilhite T Ezzelarab C Hara H Long C Ayares D Cooper DK. The Effect of Gal Expression on Pig Cells on the Human T-Cell Xenoresponse. Xenotransplantation (2012) 19(1):56–63. 10.1111/j.1399-3089.2011.00691.x 22360754 PMC3770273

[7] Abicht JM Sfriso R Reichart B Langin M Gahle K Puga Yung GL. Multiple Genetically Modified GTKO/hCD46/HLA-E/hbeta2-Mg Porcine Hearts Are Protected From Complement Activation and Natural Killer Cell Infiltration During Ex Vivo Perfusion With Human Blood. Xenotransplantation (2018) 25(5):e12390. 10.1111/xen.12390 29536572

[8] Puga Yung G Bongoni AK Pradier A Madelon N Papaserafeim M Sfriso R. Release of Pig Leukocytes and Reduced Human NK Cell Recruitment During Ex Vivo Perfusion of HLA-E/human CD46 Double-Transgenic Pig Limbs With Human Blood. Xenotransplantation (2018) 25(1). 10.1111/xen.12357 29057510

[9] Weiss EH Lilienfeld BG Muller S Muller E Herbach N Kessler B. HLA-E/human Beta2-Microglobulin Transgenic Pigs: Protection Against Xenogeneic Human Anti-Pig Natural Killer Cell Cytotoxicity. Transplantation (2009) 87(1):35–43. 10.1097/TP.0b013e318191c784 19136889

[10] Matsunami K Miyagawa S Nakai R Yamada M Shirakura R. Protection Against Natural Killer-Mediated Swine Endothelial Cell Lysis by HLA-G and HLA-E. Transplant Proc (2000) 32(5):939–40. 10.1016/S0041-1345(00)01048-4 10936284

[11] Miyagawa S Nakai R Matsunami K Kusama T Shirakura R. Co-Effect of HLA-G1 and Glycosyltransferases in Reducing NK Cell-Mediated Pig Endothelial Cell Lysis. Transpl Immunol (2003) 11(2):147–53. 10.1016/S0966-3274(02)00151-X 12799197

[12] Zhan Y Brady JL Sutherland RM Lew AM. Without CD4 Help, CD8 Rejection of Pig Xenografts Requires CD28 Costimulation But Not Perforin Killing. J Immunol (2001) 167(11):6279–85. 10.4049/jimmunol.167.11.6279 11714791

[13] Yi S Feng X Hawthorne W Patel A Walters S O’Connell PJ. CD8+ T Cells Are Capable of Rejecting Pancreatic Islet Xenografts. Transplantation (2000) 70(6):896–906. 10.1097/00007890-200009270-00007 11014643

[14] Xu XC Goodman J Sasaki H Lowell J Mohanakumar T. Activation of Natural Killer Cells and Macrophages by Porcine Endothelial Cells Augments Specific T-Cell Xenoresponse. Am J Transplant (2002) 2(4):314–22. 10.1034/j.1600-6143.2002.20405.x 12118852

[15] Seebach JD Comrack C Germana S LeGuern C Sachs DH DerSimonian H. HLA-Cw3 Expression on Porcine Endothelial Cells Protects Against Xenogeneic Cytotoxicity Mediated by a Subset of Human NK Cells. J Immunol (1997) 159(7):3655–61. 9317166

[16] Umesue M Mayumi H Nishimura Y Kong YY Omoto K Murakami Y. Donor-Specific Prolongation of Rat Skin Graft Survival Induced by Rat-Donor Cells and Cyclophosphamide Under Coadministration of Monoclonal Antibodies Against T Cell Receptor Alpha Beta and Natural Killer Cells in Mice. Transplantation (1996) 61(1):116–24. 10.1097/00007890-199601150-00023 8560549

[17] Itescu S Kwiatkowski P Artrip JH Wang SF Ankersmit J Minanov OP. Role of Natural Killer Cells, Macrophages, and Accessory Molecule Interactions in the Rejection of Pig-to-Primate Xenografts Beyond the Hyperacute Period. Hum Immunol (1998) 59(5):275–86. 10.1016/S0198-8859(98)00026-3 9619766

[18] Lin Y Vandeputte M Waer M. Natural Killer Cell- and Macrophage-Mediated Rejection of Concordant Xenografts in the Absence of T and B Cell Responses. J Immunol (1997) 158(12):5658–67. 9190914

[19] Amodio G Sales de Albuquerque R Gregori S. New Insights Into HLA-G Mediated Tolerance. Tissue Antigens (2014) 84(3):255–63. 10.1111/tan.12427 25132109

[20] Li C Houser BL Nicotra ML Strominger JL. HLA-G Homodimer-Induced Cytokine Secretion Through HLA-G Receptors on Human Decidual Macrophages and Natural Killer Cells. Proc Natl Acad Sci USA (2009) 106(14):5767–72. 10.1073/pnas.0901173106 PMC2667005 19304799

[21] Apps R Gardner L Sharkey AM Holmes N Moffett A. A Homodimeric Complex of HLA-G on Normal Trophoblast Cells Modulates Antigen-Presenting Cells *via* LILRB1. Eur J Immunol (2007) 37(7):1924–37. 10.1002/eji.200737089 PMC2699429 17549736

[22] Boyson JE Erskine R Whitman MC Chiu M Lau JM Koopman LA. Disulfide Bond-Mediated Dimerization of HLA-G on the Cell Surface. Proc Natl Acad Sci USA (2002) 99(25):16180–5. 10.1073/pnas.212643199 PMC138585 12454284

[23] Blaschitz A Juch H Volz A Hutter H Daxboeck C Desoye G. The Soluble Pool of HLA-G Produced by Human Trophoblasts Does Not Include Detectable Levels of the Intron 4-Containing HLA-G5 and HLA-G6 Isoforms. Mol Hum Reprod (2005) 11(10):699–710. 10.1093/molehr/gah185 16330474

[24] Gaynor LM Colucci F. Uterine Natural Killer Cells: Functional Distinctions and Influence on Pregnancy in Humans and Mice. Front Immunol (2017) 8:467. 10.3389/fimmu.2017.00467 28484462 PMC5402472

[25] Faas MM de Vos P. Uterine NK Cells and Macrophages in Pregnancy. Placenta (2017) 56:44–52. 10.1016/j.placenta.2017.03.001 28284455

[26] Cooper MA Fehniger TA Caligiuri MA. The Biology of Human Natural Killer-Cell Subsets. Trends Immunol (2001) 22(11):633–40. 10.1016/S1471-4906(01)02060-9 11698225

[27] Cooper MA Fehniger TA Turner SC Chen KS Ghaheri BA Ghayur T. Human Natural Killer Cells: A Unique Innate Immunoregulatory Role for the CD56(bright) Subset. Blood (2001) 97(10):3146–51. 10.1182/blood.V97.10.3146 11342442

[28] Shiroishi M Tsumoto K Amano K Shirakihara Y Colonna M Braud VM. Human Inhibitory Receptors Ig-Like Transcript 2 (ILT2) and ILT4 Compete With CD8 for MHC Class I Binding and Bind Preferentially to HLA-G. Proc Natl Acad Sci USA (2003) 100(15):8856–61. 10.1073/pnas.1431057100 PMC166403 12853576

[29] Colonna M Nakajima H Cella M. Inhibitory and Activating Receptors Involved in Immune Surveillance by Human NK and Myeloid Cells. J Leukoc Biol (1999) 66(5):718–22. 10.1002/jlb.66.5.718 10577499

[30] Rajagopalan S Long EO. A Human Histocompatibility Leukocyte Antigen (HLA)-G-Specific Receptor Expressed on All Natural Killer Cells. J Exp Med (1999) 189(7):1093–100. 10.1084/jem.189.7.1093 PMC2193010 10190900

[31] O’Callaghan CA Bell JI. Structure and Function of the Human MHC Class Ib Molecules HLA-E, HLA-F and HLA-G. Immunol Rev (1998) 163:129–38. 10.1111/j.1600-065X.1998.tb01192.x 9700506

[32] Allan DS McMichael AJ Braud VM. The ILT Family of Leukocyte Receptors. Immunobiology (2000) 202(1):34–41. 10.1016/S0171-2985(00)80050-9 10879687

[33] Colonna M Navarro F Bellon T Llano M Garcia P Samaridis J. A Common Inhibitory Receptor for Major Histocompatibility Complex Class I Molecules on Human Lymphoid and Myelomonocytic Cells. J Exp Med (1997) 186(11):1809–18. 10.1084/jem.186.11.1809 PMC2199153 9382880

[34] Naji A Menier C Morandi F Agaugue S Maki G Ferretti E. Binding of HLA-G to ITIM-Bearing Ig-Like Transcript 2 Receptor Suppresses B Cell Responses. J Immunol (2014) 192(4):1536–46. 10.4049/jimmunol.1300438 24453251

[35] Rouas-Freiss N Goncalves RM Menier C Dausset J Carosella ED. Direct Evidence to Support the Role of HLA-G in Protecting the Fetus From Maternal Uterine Natural Killer Cytolysis. Proc Natl Acad Sci USA (1997) 94(21):11520–5. 10.1073/pnas.94.21.11520 PMC23525 9326642

[36] Gonen-Gross T Goldman-Wohl D Huppertz B Lankry D Greenfield C Natanson-Yaron S. Inhibitory NK Receptor Recognition of HLA-G: Regulation by Contact Residues and by Cell Specific Expression at the Fetal-Maternal Interface. PloS One (2010) 5(1):e8941. 10.1371/journal.pone.0008941 20126612 PMC2812487

[37] Feger U Tolosa E Huang YH Waschbisch A Biedermann T Melms A. HLA-G Expression Defines a Novel Regulatory T-Cell Subset Present in Human Peripheral Blood and Sites of Inflammation. Blood (2007) 110(2):568–77. 10.1182/blood-2006-11-057125 17371944

[38] Kroemer A Xiao X Degauque N Edtinger K Wei H Demirci G. The Innate NK Cells, Allograft Rejection, and a Key Role for IL-15. J Immunol (2008) 180(12):7818–26. 10.4049/jimmunol.180.12.7818 18523245

[39] Lila N Carpentier A Amrein C Khalil-Daher I Dausset J Carosella ED. Implication of HLA-G Molecule in Heart-Graft Acceptance. Lancet (2000) 355(9221):2138. 10.1016/S0140-6736(00)02386-2 10902633

[40] Qiu J Terasaki PI Miller J Mizutani K Cai J Carosella ED. Soluble HLA-G Expression and Renal Graft Acceptance. Am J Transplant (2006) 6(9):2152–6. 10.1111/j.1600-6143.2006.01417.x 16780545

[41] Brugiere O Thabut G Krawice-Radanne I Rizzo R Dauriat G Danel C. Role of HLA-G as a Predictive Marker of Low Risk of Chronic Rejection in Lung Transplant Recipients: A Clinical Prospective Study. Am J Transplant (2015) 15(2):461–71. 10.1111/ajt.12977 25488753

[42] Pankratz S Bittner S Herrmann AM Schuhmann MK Ruck T Meuth SG. Human CD4+ HLA-G+ Regulatory T Cells Are Potent Suppressors of Graft-Versus-Host Disease In Vivo. FASEB J (2014) 28(8):3435–45. 10.1096/fj.14-251074 24744146

[43] Matsunami K Miyagawa S Nakai R Murase A Shirakura R. The Possible Use of HLA-G1 and G3 in the Inhibition of NK Cell-Mediated Swine Endothelial Cell Lysis. Clin Exp Immunol (2001) 126(1):165–72. 10.1046/j.1365-2249.2001.01622.x PMC1906174 11678914

[44] Ballinger MB Baneux PJR Barthold SW Cork LC Hau J Huerkamp MJ. In: Th, Editor. Guide for the Care and Use of Laboratory Animals. The National Academies Collection: Reports Funded by National Institutes of Health. Washington, DC (2011).

[45] Bustin SA Benes V Garson JA Hellemans J Huggett J Kubista M. The MIQE Guidelines: Minimum Information for Publication of Quantitative Real-Time PCR Experiments. Clin Chem (2009) 55(4):611–22. 10.1373/clinchem.2008.112797 19246619

[46] Hering BJ Wijkstrom M Graham ML Hardstedt M Aasheim TC Jie T. Prolonged Diabetes Reversal After Intraportal Xenotransplantation of Wild-Type Porcine Islets in Immunosuppressed Nonhuman Primates. Nat Med (2006) 12(3):301–3. 10.1038/nm1369 16491083

[47] Ihm SH Matsumoto I Sawada T Nakano M Zhang HJ Ansite JD. Effect of Donor Age on Function of Isolated Human Islets. Diabetes (2006) 55(5):1361–8. 10.2337/db05-1333 16644693

[48] Papas KK Colton CK Nelson RA Rozak PR Avgoustiniatos ES Scott WE 3rd. Human Islet Oxygen Consumption Rate and DNA Measurements Predict Diabetes Reversal in Nude Mice. Am J Transplant (2007) 7(3):707–13. 10.1111/j.1600-6143.2006.01655.x PMC2857994 17229069

[49] Papas KK Suszynski TM Colton CK. Islet Assessment for Transplantation. Curr Opin Organ Transplant (2009) 14(6):674–82. 10.1097/MOT.0b013e328332a489 PMC2859186 19812494

[50] Brandhorst H Brandhorst D Hesse F Ambrosius D Brendel M Kawakami Y. Successful Human Islet Isolation Utilizing Recombinant Collagenase. Diabetes (2003) 52(5):1143–6. 10.2337/diabetes.52.5.1143 12716744

[51] Graham ML Janecek JL Kittredge JA Hering BJ Schuurman HJ. The Streptozotocin-Induced Diabetic Nude Mouse Model: Differences Between Animals From Different Sources. Comp Med (2011) 61(4):356–60. PMC3155402 22330251

[52] Xie Z Pang D Wang K Li M Guo N Yuan H. Optimization of a CRISPR/Cas9-Mediated Knock-In Strategy at the Porcine Rosa26 Locus in Porcine Foetal Fibroblasts. Sci Rep (2017) 7(1):3036. 10.1038/s41598-017-02785-y 28596588 PMC5465212

[53] Matson AW Hosny N Swanson ZA Hering BJ Burlak C. Optimizing sgRNA Length to Improve Target Specificity and Efficiency for the GGTA1 Gene Using the CRISPR/Cas9 Gene Editing System. PloS One (2019) 14(12):e0226107. 10.1371/journal.pone.0226107 31821359 PMC6903732

[54] Li P Burlak C Estrada J Cowan P Tector J. Identification and Cloning of the Porcine ROSA26 Promoter and Its Role in Transgenesis. Transplant Technol (2014) 2:1. 10.7243/2053-6623-2-1

[55] Zhou M Hara H Dai Y Mou L Cooper DK Wu C. Circulating Organ-Specific MicroRNAs Serve as Biomarkers in Organ-Specific Diseases: Implications for Organ Allo- and Xeno-Transplantation. Int J Mol Sci (2016) 17(8):1232. 10.3390/ijms17081232 PMC5000630 27490531

[56] Watzl C. How to Trigger a Killer: Modulation of Natural Killer Cell Reactivity on Many Levels. Adv Immunol (2014) 124:137–70. 10.1016/B978-0-12-800147-9.00005-4 25175775

[57] Sullivan JA Oettinger HF Sachs DH Edge AS. Analysis of Polymorphism in Porcine MHC Class I Genes: Alterations in Signals Recognized by Human Cytotoxic Lymphocytes. J Immunol (1997) 159(5):2318–26. 9278321

[58] Burlak C Paris LL Lutz AJ Sidner RA Estrada J Li P. Reduced Binding of Human Antibodies to Cells From GGTA1/CMAH KO Pigs. Am J Transplant (2014) 14(8):1895–900. 10.1111/ajt.12744 PMC4366649 24909344

[59] Drake PM Gunn MD Charo IF Tsou CL Zhou Y Huang L. Human Placental Cytotrophoblasts Attract Monocytes and CD56(bright) Natural Killer Cells *via* the Actions of Monocyte Inflammatory Protein 1alpha. J Exp Med (2001) 193(10):1199–212. 10.1084/jem.193.10.1199 PMC2193324 11369791

[60] Mas VR Dumur CI Scian MJ Gehrau RC Maluf DG. MicroRNAs as Biomarkers in Solid Organ Transplantation. Am J Transplant (2013) 13(1):11–9. 10.1111/j.1600-6143.2012.04313.x PMC3927320 23136949

[61] Anfossi N Andre P Guia S Falk CS Roetynck S Stewart CA. Human NK Cell Education by Inhibitory Receptors for MHC Class I. Immunity (2006) 25(2):331–42. 10.1016/j.immuni.2006.06.013 16901727

[62] Lila N Amrein C Guillemain R Chevalier P Latremouille C Fabiani JN. Human Leukocyte Antigen-G Expression After Heart Transplantation Is Associated With a Reduced Incidence of Rejection. Circulation (2002) 105(16):1949–54. 10.1161/01.CIR.0000015075.89984.46 11997282

[63] Zarkhin V Talisetti A Li L Wozniak LJ McDiarmid SV Cox K. Expression of Soluble HLA-G Identifies Favorable Outcomes in Liver Transplant Recipients. Transplantation (2010) 90(9):1000–5. 10.1097/TP.0b013e3181f546af 20814356

[64] Rebmann V Bartsch D Wunsch A Mollenbeck P Golda T Viebahn R. Soluble Total Human Leukocyte Antigen Class I and Human Leukocyte Antigen-G Molecules in Kidney and Kidney/Pancreas Transplantation. Hum Immunol (2009) 70(12):995–9. 10.1016/j.humimm.2009.07.016 19651178

[65] Kaneku H. Detection of Soluble HLA-G and Its Correlation With Kidney Transplant Outcome. Clin Transpl (2006) 447–54. 18365402

[66] Crew MD Phanavanh B Garcia-Borges CN. Sequence and mRNA Expression of Nonclassical SLA Class I Genes SLA-7 and SLA-8. Immunogenetics (2004) 56(2):111–4. 10.1007/s00251-004-0676-z 15118849

[67] Ravetch JV Lanier LL. Immune Inhibitory Receptors. Science (2000) 290(5489):84–9. 10.1126/science.290.5489.84 11021804

[68] Boyson JE Iwanaga KK Golos TG Watkins DI. Identification of a Novel MHC Class I Gene, Mamu-AG, Expressed in the Placenta of a Primate With an Inactivated G Locus. J Immunol (1997) 159(7):3311–21. 9317129

[69] Bondarenko GI Burleigh DW Durning M Breburda EE Grendell RL Golos TG. Passive Immunization Against the MHC Class I Molecule Mamu-AG Disrupts Rhesus Placental Development and Endometrial Responses. J Immunol (2007) 179(12):8042–50. 10.4049/jimmunol.179.12.8042 PMC6191312 18056344

[70] Bondarenko GI Dambaeva SV Grendell RL Hughes AL Durning M Garthwaite MA. Characterization of Cynomolgus and Vervet Monkey Placental MHC Class I Expression: Diversity of the Nonhuman Primate AG Locus. Immunogenetics (2009) 61(6):431–42. 10.1007/s00251-009-0376-9 PMC2810720 19468726

[71] Slukvin II Grendell RL Rao DS Hughes AL Golos TG. Cloning of Rhesus Monkey LILRs. Tissue Antigens (2006) 67(4):331–7. 10.1111/j.1399-0039.2006.00579.x 16634871

[72] Ristich V Zhang W Liang S Horuzsko A. Mechanisms of Prolongation of Allograft Survival by HLA-G/ILT4-Modified Dendritic Cells. Hum Immunol (2007) 68(4):264–71. 10.1016/j.humimm.2006.11.008 17400062

[73] Ketroussi F Giuliani M Bahri R Azzarone B Charpentier B Durrbach A. Lymphocyte Cell-Cycle Inhibition by HLA-G Is Mediated by Phosphatase SHP-2 and Acts on the mTOR Pathway. PloS One (2011) 6(8):e22776. 10.1371/journal.pone.0022776 21887223 PMC3160837

[74] Kapasi K Albert SE Yie S Zavazava N Librach CL. HLA-G has a Concentration-Dependent Effect on the Generation of an Allo-CTL Response. Immunology (2000) 101(2):191–200. 10.1046/j.1365-2567.2000.00109.x 11012772 PMC2327080

[75] Lorenz U. SHP-1 and SHP-2 in T Cells: Two Phosphatases Functioning at Many Levels. Immunol Rev (2009) 228(1):342–59. 10.1111/j.1600-065X.2008.00760.x PMC2669678 19290938

[76] Esquivel EL Maeda A Eguchi H Asada M Sugiyama M Manabe C. Suppression of Human Macrophage-Mediated Cytotoxicity by Transgenic Swine Endothelial Cell Expression of HLA-G. Transpl Immunol (2015) 32(2):109–15. 10.1016/j.trim.2014.12.004 25559170

[77] Liang S Ristich V Arase H Dausset J Carosella ED Horuzsko A. Modulation of Dendritic Cell Differentiation by HLA-G and ILT4 Requires the IL-6–STAT3 Signaling Pathway. Proc Natl Acad Sci USA (2008) 105(24):8357–62. 10.1073/pnas.0803341105 PMC2448841 18550825

