# Supplementary material for: HLA-G1+ Expression in GGTA1KO Pigs Suppresses Human and Monkey Anti-Pig T, B and NK Cell Responses
Source: Front Immunol. 2021 Sep 9;12:730545. doi: 10.3389/fimmu.2021.730545 (PMC8459615; doi:10.3389/fimmu.2021.730545)
Supplement: Supplementary Figure 1 — HLA-G1 integration in the porcine ROSA26 locus. (A) Sanger sequence product of GTKO/HLA-G1 piglet (B) Next generation sequence of HLA-G1+. [file DataSheet_1.pdf]

Supplemental Figure 1

A

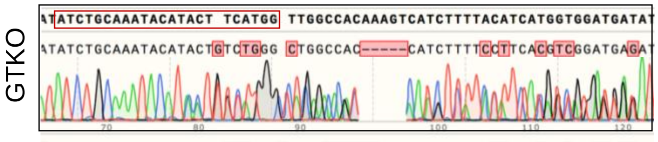

-Genomic DNA GGTA1 gene sequence

-Sanger sequence product from GTKO/HLA-G1 piglet

B

Next generation sequence confirmation of HLA-G1⁺ piglets:

CAAGTAAAAGGTAAACGTGGAGTAGGCAGTTCCCAGGCTAGGGGTTGAATAGGCGTGGGGGGAGGGGAAGAGTCCTGACC
CAGGGAAGACATTAAAAAGGTAGTGGGGTCGACTAGATGAAGGAGAGCCTTTCTCTCTGGGCAAGAGCGGTGCAATGGTGT
GTAAAGGTAGCTGAGAAGACGAAAAGGGCAAGCATCTTCCTGCTACCAGGCTGGGGAGGCCCAGGCCCACGACCCCGAGGA
GAGGGAACGCAGGGAGACTGAGGTGACCCTTCTTTCCCCCGGGGCCCGGTCGTGTGGTTCGGTGTCTCTTTTCTGTTGGACCC
TTACCTTGACCCAGGCGCTGCCGGGGCCTGGGACCCAGGGCCAGCTCGCCGCCAATCAGCAGTGTGGTACTTTGTCTTGAGGA
GATGTCCTGGACTCACACGGAAACTTAGGGCTACGGAATGAAGTTCTCACTCCCATTAGGTGACAGGTTTTTAGAGAAGCCAA
TCAGCGTCGCCGCGGTCCTGGTTCTAAAGTCCTCGCTCACCCACCCGGACTCATTCTCCCCAGACGCCAAGGATGGTGGTCATG
GCGCCCCGAACCCTCTTCCTGCTGCTCTCGGGGGCCCTGACCCTGACCGAGACCTGGGCGGGCTCCCACTCCATGAGGTATTTC
AGCGCCGCCGTGTCCCGGCCCGGCCGCGGGGAGCCCCGCTTCATCGCCATGGGCTACGTGGACGACACGCAGTTCGTGCGGT
TCGACAGCGACTCGGCGTGTCCGAGGATGGAGCCGCGGGCGCCGTGGGTGGAGCAGGAGGGGCCGGAGTATTGGGAAGAG
GAGACACGGAACACCAAGGCCCACGCACAGACTGACAGAATGAACCTGCAGACCCTGCGCGGCTACTACAACCAGAGCGAG
GCCAGTTCTCACACCCTCCAGTGGATGATTGGCTGCGACCTGGGGTCCGACGGACGCCTCCTCCGCGGGTATGAACAGTATGC
CTACGATGGCAAGGATTACCTCGCCCTGAACGAGGACCTGCGCTCCTGGACCGCAGCGGACACTGCGGCTCAGATCTCCAAGC
GCAAGTGTGAGGCGGCCAATGTGGCTGAACAAAGGAGAGCCTACCTGGAGGGCACGTGCGTGGAGTGGCTCCACAGATACC
TGGAGAACGGGAAGGAGATGCTGCAGCGCGCGGACCCCCCCAAGACACACGTGACCCACCACCCTGTCTTTGACTATGAGGC
CACCCTGAGGTGCTGGGCCCTGGGCTTCTACCCTGCGGAGATCATACTGACCTGGCAGCGGGATGGGGAGGACCAGACCCAG
GACGTGGAGCTCGTGGAGACCAGGCCTGCAGGGGATGGAACCTTCCAGAAGTGGGCAGCTGTGGTGGTGCCTTCTGGAGAG
GAGCAGAGATACACGTGCCATGTGCAGCATGAGGGGCTGCCGGAGCCCCTCATGCTGAGATGGAAGCAGTCTTCCCTGCCCA
CCATCCCCATCATGGGTATCGTTGCTGGCCTGGTTGTCCTTGCAGCTGTAGTCACTGGAGCTGCGGTCGCTGCTGTGCTGTGGA
GAAAGAAGAGCTCAGATTGAAAAGGAGGGAGCTACTCTCAGGCTGCAGAGACCAGCCCACCCTGTGCCACCATGACCCTCTTC
CTCATGCTGAACTGCATTCCTTCCCCAATCACCTTTCCTGTTCCAGAAAAGGGGCTGGGATGTCTCCGTCTCTGTCTCAAATTTG
TGGTCACTGAGCTATAACTTACTTCTGTATTAAAATTAGAATCTGAGTATAAATTTACTTTTTCAAATTATTTCCAAGAGAGATTG
ATGGGTTAATTAAAGGAGAAGATTCCTGAAATTTGAGAGACAAAATAAATGGAAGACATGAGAACTTTCACCCTGCAGTTGGA
AAGGGAACTATTTCGGCTTTGGGGCTCCGGCTCCTCAGAGAGCCTCGGCTAGGTAGGGGAGCGGGACTCTGGTTTGGGGGA
GGGCCGGCGGTTTGGCGGGGGATGGGTGCTTGAGGTGGTCTGACCGGTAGCGGGGGTCGCCTTCCCTAGCGGGAAGTCGGG
AGCATATCGTTTGTTACGCTGGAAGGGGAAGAGGTGGTGAGAGGCAGGCGGGAGTGCGGCCCGCCCTGCGGCAACCGGAG
GGGGAGGGAGAAGGGAGCGGAAAAGCCTGGAATACGGACGGAGCCATTGCTCCCGCAGAGGGAGGGGAGGAGCGCTTCCT
GCTCTTCTCTTGTCACTGATTGGCCGCTTCTCCTCCCGCCGTGTGTGAAAACACAAATGGCGTGTTTTGGTTGGAGTAAAGCTCC
TGTCAGTTACAGCCTCGGGAGTGCGCAGCCTCCCAGGAACTCTCGCATTGCCCCCTGGGTGGGTAGGTAGGTGGGGTGGAGA
GAGCTGCACAGGCGGGCGCTGTCGGCCTCCTGCGGGGGGAGGGGAGGGTCAGTGAAAGTGGCTCCCGCGCGGGCGTCCTG
CCACCCTCCCCTCCGGGGGAGTCGGTTTACCCGCCGCCTGCTCGGCTTTGGTATCTGATTGGCTGCTGAAGTCCTGGGAACGG
CCCCTTGTTATTGGCTTGGGTCCCAAATGAG
