# Supplementary figures and images for: HLA-G1+ Expression in GGTA1KO Pigs Suppresses Human and Monkey Anti-Pig T, B and NK Cell Responses
Source: Front Immunol. 2021 Sep 9;12:730545. doi: 10.3389/fimmu.2021.730545 (PMC8459615; doi:10.3389/fimmu.2021.730545)

**Supplemental Figure 2.** Confocal analysis of WT adult porcine tissue samples

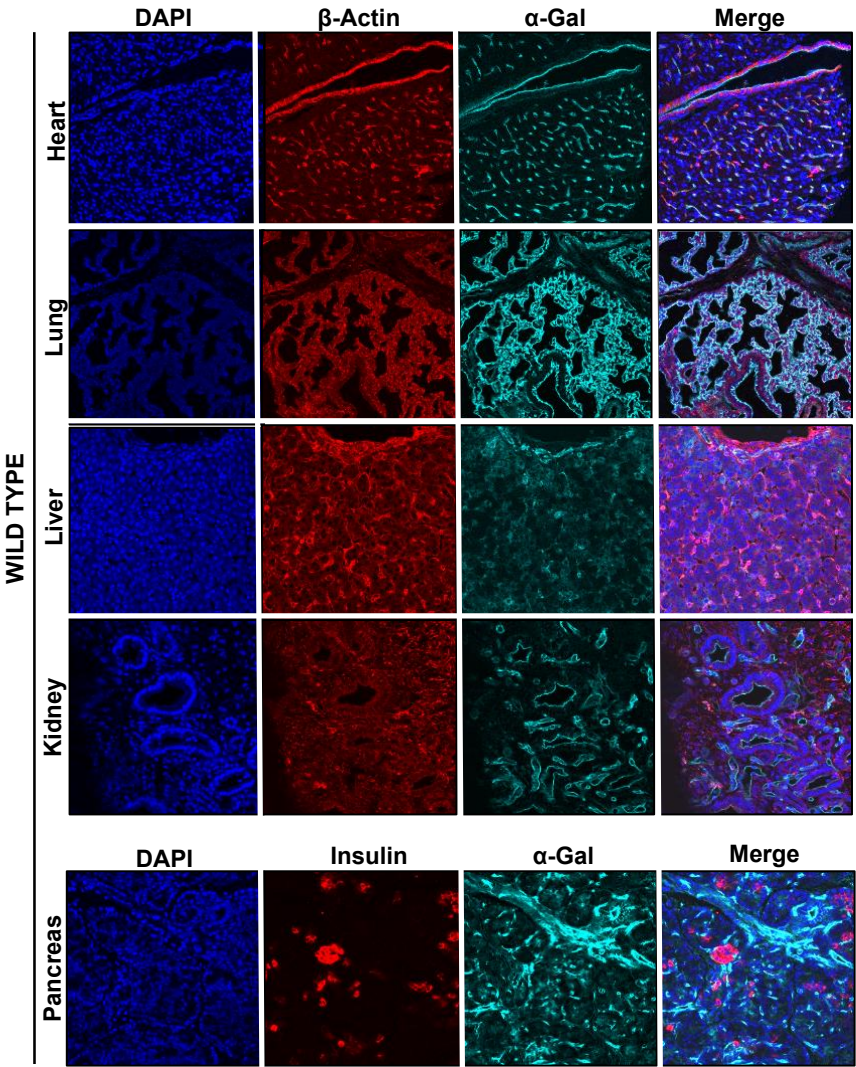

Supplement: Supplementary Figure 2 — Confocal analysis of WT porcine tissue samples. WT heart, lung, liver, kidney and pancreas were paraffin embedded, sectioned and labelled with Rabbit mAb to b-actin [13E5] (Cell Signaling Technology; #4970S) and tagged to Goat anti-rabbit IgG AF 488 (Invitrogen; A11008), Isolectin GS-IB4 AF 647 Conjugate (Life Technologies; I32450), Mouse HLA-G monoclonal Antibody [87G] (Invitrogen; MA1-10356) and tagged to Goat anti-mouse IgG AF 555 (Invitrogen; A32727). Goat anti-mouse IgG1 Fc Secondary Antibody FITC (Invitrogen; 31547) was used as an isotype control for 87G. Pancreas section were labelled with Polyclonal Guinea Pig Anti-insulin (Dako; A0564) tagged to Donkey Anti-guinea pig IgG (H+L) AF 647 (Jackson Immuno Research; 706-606-148) instead of b-actin. DAPI was used to label nuclei of all cells. All sections were imaged using the 10X, 20X and 40X objective on Olympus Fluoview 3000 inverted confocal microscope. [file DataSheet_2.pdf]
