# Supplementary material for: HLA-G1+ Expression in GGTA1KO Pigs Suppresses Human and Monkey Anti-Pig T, B and NK Cell Responses
Source: Front Immunol. 2021 Sep 9;12:730545. doi: 10.3389/fimmu.2021.730545 (PMC8459615; doi:10.3389/fimmu.2021.730545)
Supplement: Supplementary Table 1 — Quantitative PCR probe sequences and product size. [file Table_1.pdf]

Supplemental Table 1

Genes	Primer sequence (5'-3')	PCR Product Size (bp)	Tm (°C)	Reference/Sequence Accession No.
Pig ACTB F	AGATCGTGCGGGACATCAAG	73	60	XM_021086047.1
Pig ACTB R	GCGGCAGTGGCCATCTC	73	60	XM_021086047.1
Human ACTB	GATGAGATTGGCATGGCTTTATT	99	60	NM_001101.5
Human ACTB	ACCTTCACCCGTTCCAGTTT	99	60	NM_001101.5
Human HLA-G	CTCCCACTCCATGAGGTATTTC	478	60	NM_001363567.2
Human HLA-G	CAGGTAGGCTCTCCTTTGTTC	478	60	NM_001363567.2
